# Positron Emission Tomography Imaging of Bacterial Infections With an Enterobactin Analog to Monitor Treatment Efficacy With a Catechol Antibiotic

**DOI:** 10.1002/anie.2391261

**Published:** 2026-03-11

**Authors:** M. Andrey Joaqui‐Joaqui, Phuong Nguyen Tran, Axia Marlin, Fiona Armstrong‐Pavlik, Minhua Cao, Valérie C. Pierre, Eszter Boros

**Affiliations:** ^1^ Department of Chemistry University of Wisconsin – Madison Madison Wisconsin USA; ^2^ Department of Chemistry University of Minnesota Minneapolis Minnesota USA; ^3^ Department of Chemistry University of Utah Salt Lake City UT USA

**Keywords:** cefiderocol, enterobactin, imaging, infection, radiotracer, siderophore

## Abstract

Positron emission tomography (PET) is an emerging tool under clinical investigation for the detection of bacterial infections. The ability to identify the site of infection and the pathogen's potential susceptibility to subsequent treatment approaches can streamline treatment and prevent misuse of antibiotics. Previous studies identified TREN‐CAM as a promising scaffold for radiometal labeling using the positron‐emitting radionuclide ^68^Ga. Here, we investigate the suitability of ^67/68^Ga‐labeled TREN‐CAM and enterobactin for the noninvasive imaging of an *Escherichia coli* infection. In vitro uptake experiments with [^67^Ga]Ga^III^‐TREN‐CAM demonstrate superior uptake in *E. coli* K12 and *Pseudomonas aeruginosa* PAO1 when compared with [^67^Ga]Ga^III^‐DFO, a compound currently under clinical investigation for infection imaging. Selectivity experiments indicate that [^67^Ga]Ga^III^‐TREN‐CAM is recognized by the active transmembrane transport machinery for ^nat^Fe^III^‐Ent. Using a soft tissue *E. coli* infection model, we demonstrate that [^68^Ga]Ga^III^‐TREN‐CAM selectively accumulates in infected tissue. Notably, [^68^Ga]Ga^III^‐TREN‐CAM provides better infection‐to‐inflammation contrast, accelerated blood clearance, and lower off‐target accumulation compared to [^68^Ga]Ga^III^‐Ent and [^68^Ga]Ga^III^‐citrate. The potential of [^68^Ga]Ga^III^‐TREN‐CAM to serve as a tool to monitor the therapeutic efficacy of cefiderocol is also evaluated, offering insight into its utility in guiding antibiotic treatment.

## Graphical abstract

**Synopsis**. The [^68^Ga]Ga^III^‐TREN‐CAM radiochelate selectively accumulates in Gram‐negative sites of infection and noninvasively monitors treatment response a siderophore antibiotic.

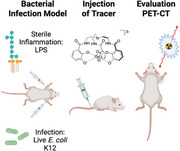

## 1 Introduction

Despite the increasing burden of bacterial infections on our society and the rise of antibiotic resistance, bacterial infections are diagnosed primarily by physical exam, and initial treatment often involves empiric use of broad‐spectrum antibiotics [1, 2, 3]. Proper identification of the causative pathogen requires in vitro molecular biology and microbiology assays that rely on biopsy of the suspected site of infection followed by successful culturing [4, 5]. The typical success rate of culture and identification of pathogens from biopsy is low [6]; the lack of success and time scale of unequivocal identification incompatible with rapidly progressing, systemic infections and sepsis results in prophylactic, systemic treatment of patients with broad‐spectrum antibiotics. Such misuse of antibiotics enhances emergence of resistance, resulting in a growing global health concern.

**FIGURE 1:**
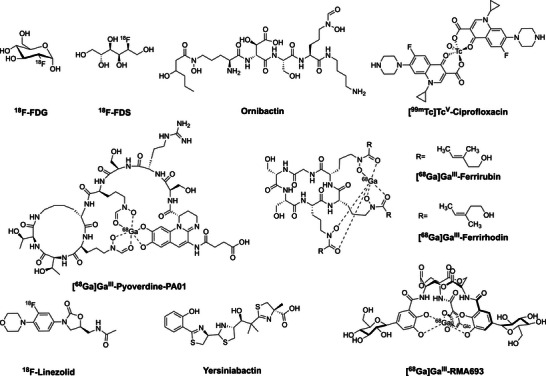
Structures of select radiotracers previously investigated for the imaging of bacterial infections in vivo.

Therefore, there is a need for methods that enable localization and identification of the bacterial infection noninvasively and rapidly to inform treatment and diminish misuse of antibiotics. Nuclear imaging techniques are ideally suited for this purpose. However, to date, no noninvasive, diagnostic agents selective for bacterial infection are approved for clinical use in humans. [^67^Ga]Ga‐citrate has been clinically approved to image infections with single photon emission computed tomography (SPECT); however, it lacks spatial resolution and specificity for infection over inflammation. The positron emission tomography (PET) agent most commonly used to image bacterial infection remains [^18^F]‐fluorodeoxyglucose (^18^FDG, Figure 1) [7, 8]. This tracer, however, does not distinguish between infection and sterile inflammation, as its uptake mechanism relies on a host‐mediated inflammation response rather than direct uptake by the bacterial pathogen [9]. Other PET tracers are under active investigation for bacterial imaging. 2‐[^18^F]‐Fluorodeoxysorbitol (^18^F‐FDS) has shown higher specificity than ^18^F‐FDG in differentiating a *Klebsiella pneumoniae* lung infection from lung inflammation in mice [10]. ^18^F‐Linezolid [11] has been tested in a preclinical pneumonic‐tuberculosis mouse infection model but showed poor specificity, with similar uptake in infected and healthy lung tissue. Other approaches that exploit differences in metabolism between bacteria and mammals include the use of ^11^C‐ and ^18^F‐labeled D‐amino acids [12, 13, 14, 15] and *p*‐aminobenzoic acid (PABA) analogs [16, 17, 18]. The former take advantage of the incorporation of D‐amino acids into bacterial peptidoglycan to selectively target bacteria, whereas PABA‐based tracers target the bacterial folate biosynthesis pathway. Antibiotics‐based tracers, such as [^99m^Tc]Tc^V^‐ciprofloxacin (Figure 1) have been evaluated in both rodent models and clinical trials in humans; however, it showed poor selectivity and produced false negatives [19, 20, 21, 22]. The use of other antibiotic‐derived radiotracers for PET imaging of bacterial infections has shown limited utility due to high nonspecific/off‐target uptake or unfavorable pharmacokinetics [9].

An alternative strategy for targeted in vivo imaging of bacterial infections exploits siderophores, low molecular weight iron chelators that are well‐known virulence factors for pathogenic bacteria [23, 24, 25, 26, 27]. Bacteria and fungi recognize and actively transport ferric siderophore complexes via cell‐surface receptors that are absent in mammalian cells [28]. The similarity of Ga^III^ and Fe^III^ coordination chemistry (6‐coordinate ionic radius for Ga^3+^ = 0.620 Å vs 0.645 Å for Fe^3+^), results in complexes with close structural homology, promoting the selective uptake of Ga^III^ siderophore complexes [29, 30]. This is particularly advantageous for PET imaging, since the ^68^Ga radioisotope exhibits ideal emission properties for imaging probe development (Eβ_+avg_ = 0.83 MeV, 89%, *t*_1/2_ = 68 min) [31]. Indeed, the imaging of bacterial infections with ^68^Ga‐siderophore complexes has been explored extensively in this regard: [^68^Ga]Ga^III^‐triacetylfusarinine C demonstrated selective in vivo localization at sites of pulmonary *Aspergillus fumigatus* infections [32], [^68^Ga]Ga^III^‐pyoverdine demonstrated feasibility of imaging *Pseudomonas aeruginosa* in the lung and soft tissue [33]. Other recent examples of bacteria‐specific PET imaging agents include reports of ^68^Ga‐labeled siderophores, such as ornibactin‐C6 [34], ferrirubin, and ferrirhodin [35], as well as a ^64^Cu‐labeled yersiniabactin [36]. [^68^Ga]Ga^III^‐desferrioxamine ([^68^Ga]Ga^III^‐DFO) has demonstrated selective accumulation in *P. aeruginosa* and *Staphylococcus aureus* preclinical infection animal models [33]. Recently, we showed that [^68^Ga]Ga^III^‐DFO‐linked antibiotic drug conjugates also effectively localize at sites of infection [37]. [^68^Ga]Ga^III^‐DFO has recently entered clinical trials to evaluate its potential as a PET imaging agent for vascular graft infections (Clinical trial: NCT05285072).

However, despite the significant body of published work in this area, siderophore catechol‐based radiotracers remain underexplored for in vivo PET imaging of Gram‐negative pathogens, such as *P. aeruginosa* and *Escherichia coli*. A few recent examples are the use of the salmochelin derivative [^68^Ga]Ga‐RMA693 to image the *E. coli* strain ATCC 25922 [38] and use of a catechol‐functionalized, tetrapodal 1,4,7,10‐tetraazacyclododecane‐1,4,7,10‐tetraacetic amide (DOTAM) siderophore mimic, linked to  1,4,7,10‐tetraazacyclododecane‐1,4,7,10‐tetraacetic acid (DOTA) to incorporate ^68^Ga^III^ [39]. While [^68^Ga]Ga‐RMA693 effectively differentiates bacterial infection from sterile inflammation, its vulnerability to nucleophiles and oxidants due to the macrolactone ring remains a significant limitation. DOTAM conjugates require distinct pH and high‐temperature conditions for ^68^Ga^III^ incorporation, which restricts their application from being employed using a shake‐and‐shoot radiopharmaceutical preparation kit. Nonetheless, their ability to differentiate infection from inflammation further highlights their potential. A siderophore‐based agent featuring catechol units that resists degradation and allows rapid ^68^Ga^III^ complexation under mild conditions is therefore highly desirable for clinical translation.

**FIGURE 2:**
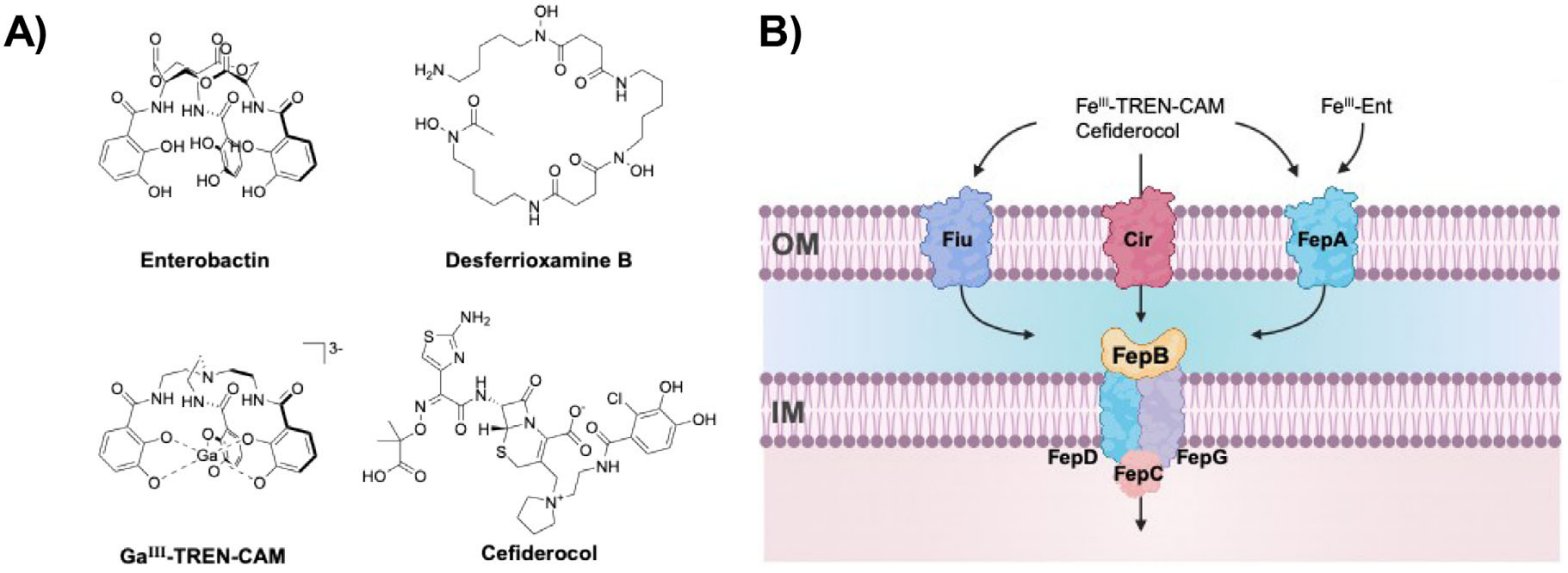
(A) Chemical structures of cefiderocol, desferrioxamine (DFO), enterobactin (Ent), and its corresponding Ga‐siderophore mimic Ga^III^‐TREN‐CAM. (B) Possible catecholate siderophore transport systems in E. coli.

Enterobactin, a catecholate siderophore, and its synthetic mimic TREN‐CAM exhibit high affinity for iron(III), as well as gallium(III) [40, 41]. In TREN‐CAM, the macrolactone backbone characteristic of enterobactin is replaced by a tripodal amine cap to reduce susceptibility to hydrolysis. The recognition and transmembrane transport of ferric enterobactin and Fe^III^‐TREN‐CAM by a wide range of Gram‐negative and Gram‐positive bacteria opens the possibility of imaging bacterial pathogens within the mammalian host with a ^68^Ga‐labeled analogue. Furthermore, the mono‐catechol‐functionalized antibiotic cefiderocol (Figure 2), which has recently gained FDA approval, utilizes the same family of transmembrane transporters [42, 43, 44, 45, 46, 47]. Thus, a catechol‐based PET agent would not only provide identification of the site of infection but could also stratify patients for treatment with cefiderocol.

Previously, we have demonstrated that [^68^Ga]Ga^III^‐TREN‐CAM (Figure 2) is suitable for in vivo applications. Here, we synthesize and evaluate [^68^Ga]Ga^III^‐TREN‐CAM in direct comparison with [^68^Ga]Ga^III^‐DFO for selective uptake in both Gram‐negative (*P. aeruginosa and E. coli*) and Gram‐positive (*S. aureus*) pathogens. Likewise, their potential to accumulate selectively at a site of infection in vivo using PET imaging and biodistribution analysis is explored and compared to that of [^68^Ga]Ga^III^‐citrate, a tracer commonly used for imaging of inflammation and bacterial infections. Subsequently, the potential of [^68^Ga]Ga^III^‐TREN‐CAM as a diagnostic tool to predict and monitor the therapeutic efficacy of cefiderocol is evaluated, offering insight into its utility in guiding antibiotic treatment.

## 2 Results and Discussion

### 2.1 Radiochemistry and Bacterial Uptake Studies

Nonradioactive reference Ga^3+^ complexes of TREN‐CAM and DFO were synthesized as previously reported in the literature [48, 49]. Ga^3+^ and Fe^3+^ complexes of enterobactin were prepared in a similar manner with successful synthesis determined by ^1^H NMR, RP‐HPLC, UV–vis spectroscopy, and HR‐ESI‐MS (Figures S1–S7).

To evaluate the ability of [^68^Ga]Ga^III^‐TREN‐CAM and [^68^Ga]Ga^III^‐Ent to selectively accumulate in bacteria, we first sought to optimize radiolabeling conditions to form radiochelates with high radiochemical purity and conversion. We employed ^68^Ga (*t*_1/2_ = 68 min) and ^67^Ga (*t*_1/2_ = 79 h) [31], the long‐lived congener of ^68^Ga, to conduct subsequent in vitro experiments.

**FIGURE 3:**
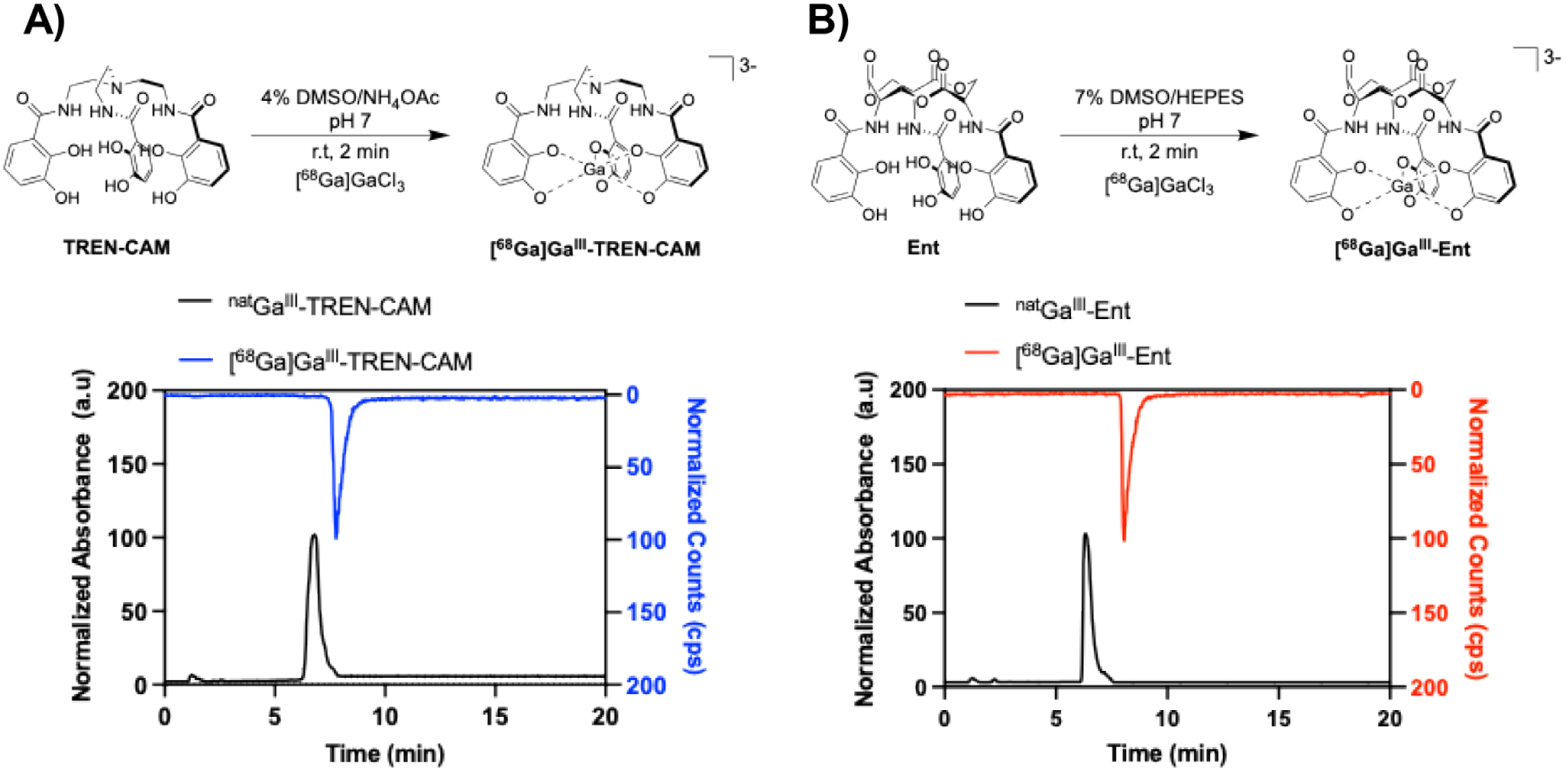
Scheme for the optimized radiosynthesis and chromatographic analysis of ^68^Ga‐labeled tracers and their corresponding nonradioactive complexes. (A) [^68^Ga]Ga^III^‐TREN‐CAM. (B) [^68^Ga]Ga^III^‐Ent.

Optimization of radiolabeling conditions was conducted with both radioisotopes, using ^67/68^GaCl_3_ (formally, but likely present under high dilution, acidic conditions as the 6‐coordinate aqua ion). [^68^Ga]Ga^III^‐TREN‐CAM and [^68^Ga]Ga^III^‐Ent were radiolabeled at room temperature and pH 7 in 2 min, achieving a molar activity of 33 mCi/µmol and 3 mCi/µmol with a > 95% radiochemical yield (Figure 3), respectively. Given the high radiochemical purity of the resulting radiotracers (Figures S8–S11), no additional purification or isolation processes were required prior to subsequent in vitro or in vivo studies, indicating compatibility with prospective, kit‐type radiopharmaceutical preparation.

The ability of Ga^III^‐TREN‐CAM to function as an efficient surrogate of ferric enterobactin for bacterial imaging was first evaluated in vitro with the radiolabeled complex [^67^Ga]Ga^III^‐TREN‐CAM. Recognition and association of the probe were assessed with *E. coli* K12, *P. aeruginosa* PAO1, and *S. aureus* RN4220. Briefly, cultures of each bacterial strain that had reached the desired OD_600_ were incubated with 10 µCi of [^67^Ga]Ga^III^‐TREN‐CAM in iron‐deficient media, and quantification of bacterial uptake was conducted at 10, 20, 30, 60, and 120 min by pelleting and washing to ensure removal of nonbound ^67^Ga tracer. While this assay does not distinguish between peri‐ and cytoplasmic uptake, it quantifies selective association of the ^67^Ga‐complex with the bacteria.

**FIGURE 4:**
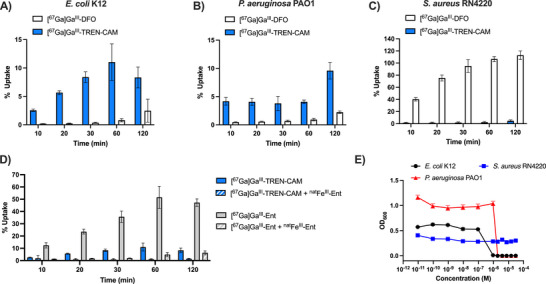
Time dependent, radiochemical bacterial uptake of [^67^Ga]Ga^III^‐TREN‐CAM and [^67^Ga]Ga^III^‐DFO in Gram‐negative and Gram‐positive strains; (A) *E. coli* K12; (B) *P. aeruginosa* PAO1; (C) *S. aureus* RN4220. D) Time‐dependent, radiochemical bacterial uptake competition assays for [^67^Ga]Ga^III^‐TREN‐CAM and [^67^Ga]Ga^III^‐Ent in *E. coli* K12 in presence of 200x ^nat^Fe^III^‐Ent. (E) Antibacterial activity of cefiderocol against *E. coli* K12, *P. aeruginosa* PAO1, and *S. aureus* RN4220, in MHB iron‐deficient medium, 18 h, 37 °C, *n*=9, and mean ± SD.

We quantified the uptake of [^67^Ga]Ga^III^‐DFO, which is internalized predominantly by Gram‐positive strains as a comparative benchmark [33]. As illustrated in Figure 4a–c and Tables S2–S4, [^67^Ga]Ga^III^‐TREN‐CAM rapidly associates with all tested bacterial strains in vitro, reaching average uptake values of 8.33 ± 1.83% in *E. coli* K12 and 9.60 ± 1.47% in *P. aeruginosa* PAO1 after 120 min. Compared to [^67^Ga]Ga^III^‐DFO, [^67^Ga]Ga^III^‐TREN‐CAM demonstrates markedly higher uptake in both Gram‐negative organisms, with more than three‐fold higher uptake in *E. coli* K12 (2.47 ± 2.02% DFO uptake) and four‐fold *in P. aeruginosa* PAO1 (2.23 ± 0.22% DFO uptake) within the first 2 h. Conversely, in *S. aureus* RN4220, [^67^Ga]Ga^III^‐TREN‐CAM shows moderate uptake (4.69 ± 1.58%) relative to [^67^Ga]Ga^III^‐DFO (112.98 ± 7.09%). The substantial uptake of [^67^Ga]Ga^III^‐DFO in *S. aureus* RN4220 is consistent with Petrik's findings, which showed high uptake of this hydroxamate‐type siderophore in multiple *S. aureus* strains, with no notable accumulation in a wide variety of *E. coli* strains. [33] In addition to uptake studies in *E. coli* K12 and *P. aeruginosa*, we extended our evaluation to include Gram‐negative pathogens of the ESKAPE family (*K. pneumoniae, Enterobacter cloacae*, and *Acinetobacter baumannii*) using the nonradioactive complexes ^nat^Ga^III^‐TREN‐CAM and ^nat^Ga^III^‐DFO similarly in iron‐deficient media (Table S1). The results (Figures S12 and S13) showed a consistent trend, with TREN‐CAM exhibiting higher uptake than DFO across all tested strains.

Given the similarity in coordination chemistry between Ga^III^ and Fe^III^, we hypothesize that [^67^Ga]Ga^III^‐TREN‐CAM can accumulate in bacteria utilizing the same active transmembrane transport mechanisms as ferric enterobactin (FepA) and other catechol‐based siderophores and their synthetic mimics (Cir, Fiu), Figure 2b. *P. aeruginosa* does not secrete enterobactin but possesses outer‐membrane TonB‐dependent transporters TBDT and PfeA, capable of transporting catechol‐based siderophores into the periplasm. [50, 51] Similarly, *S. aureus* does not produce catechol‐type siderophores, though they can be recognized and transported by ABC transporters encoded by the sstABCD (staphylococcal siderophore transporter) operons [52, 53]. Literature reports also indicate that *K. pneumoniae, Enterobacter cloacae*, and *Acinetobacter baumannii* can utilize enterobactin to circumvent iron limitation, underscoring the use of TREN‐CAM as an effective enterobactin mimic. [25, 54, 55] Regardless of the incubation time, [^67^Ga]Ga^III^‐TREN‐CAM accumulates in *E. coli* K12 and *P. aeruginosa* PAO1 at significantly higher levels than [^67^Ga]Ga^III^DFO. Similar results were observed when evaluating the uptake of the nonradioactive complexes ^nat^Ga^III^‐TREN‐CAM and ^nat^Ga^III^‐DFO in *E. coli* K12 (Figure S13), highlighting the superior capabilities of the catechol‐based probe to localize in Gram‐negative organisms.

In addition to the time‐dependent uptake of [^67^Ga]Ga^III^‐TREN‐CAM in *E. coli* K12, assays were conducted by incubation of excess Fe^III^‐Ent before addition of [^67^Ga]Ga^III^‐TREN‐CAM. These challenge assays probed the selectivity of [^67^Ga]Ga^III^‐TREN‐CAM for the Fe^III^‐Ent uptake machinery; incubation with Fe^III^‐Ent saturates transmembrane transporters and down‐regulates their expression. [56] This experiment was also carried out with ^6^ ^7^Ga‐labeled enterobactin in order to provide a reference system. As expected, [^67^Ga]Ga^III^‐Ent demonstrates robust, time‐dependent uptake by *E. coli* K12, which is substantially diminished in the presence of excess native siderophore, ^nat^Fe^III^‐Ent (Figure 4d). Similarly for [^67^Ga]Ga^III^‐TREN‐CAM, co‐incubation with ^nat^Fe^III^‐Ent significantly inhibited its time‐dependent uptake, supporting the hypothesis of competitive inhibition at siderophore transporters and suggesting that [^67^Ga]Ga^III^‐TREN‐CAM is internalized via the same bacterial uptake pathways as ^nat^Fe^III^‐Ent.

Cefiderocol, a recently FDA‐approved cephalosporin antibiotic that incorporates a catechol moiety, utilizes the outer membrane receptors FepA, CirA, and Fiu to enter bacterial cells (Figure 2b) in direct analogy with Fe^III^/Ga^III^‐TREN‐CAM. Indeed, we observed good correlation between the percentage of bacterial uptake of [^67^Ga]Ga^III^‐TREN‐CAM and the antibacterial potency of cefiderocol against Gram‐negative strains (Figure 4e). Cefiderocol does not inhibit the growth of *S. aureus* RN4220 (MIC_98_> 30 µM) and shows lower uptake of [^67^Ga]Ga^III^‐TREN‐CAM (4.69 ± 1.58%). In contrast, *E. coli* K12 and *P. aeruginosa* PAO1, which exhibit an uptake of 8.33 ± 1.83% and 9.60 ± 1.47%, respectively, have cefiderocol MIC_98_ values of 1 and 2 µM, respectively.

These findings suggest that imaging bacterial infections in vivo via PET using [^68^Ga]Ga^III^‐TREN‐CAM could offer a dual advantage: (1) provide a noninvasive diagnostic tool to localize bacterial infections and (2) function as a predictive marker for cefiderocol susceptibility. This provides an approach to directly stratify patients for therapy with cefiderocol following a positive [^68^Ga]Ga^III^‐TREN‐CAM scan and monitor treatment efficacy.

### 2.2 Naïve in Vivo Biodistribution of Ga^III^‐TREN‐CAM and Ga^III^‐Ent

**FIGURE 5:**
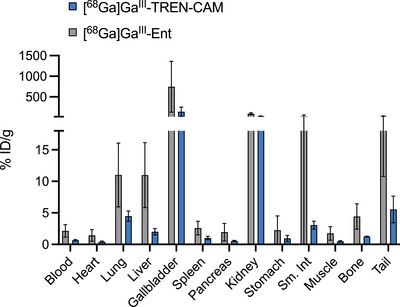
Ex vivo biodistribution pattern of [^68^Ga]Ga^III^‐TREN‐CAM and [^68^Ga]Ga^III^‐Ent 1.5 h post injection. Error bars represent ±1 standard deviation (*n* = 4). % ID/g of organ is the percentage of injected dose per gram of organ weight.

To further evaluate the potential of [^68^Ga]Ga^III^‐TREN‐CAM as a bacteria‐specific imaging agent for infections, we examined its in vivo biodistribution profile in healthy BALB/c mice (*n* = 4) in direct comparison with [^68^Ga]Ga^III^‐Ent. As shown in Figure 5 and Table S5, 90 min post injection of radiotracers formulated in DPBS buffer (Figure S14), a major fraction of [^68^Ga]Ga^III^‐Ent is found in the kidneys (90.53 ± 26.55%ID/g), consistent with rapid renal clearance. High levels of the radiotracer were also observed in soft tissues, such as liver (10.99 ± 5.15%ID/g), gallbladder (745.71 ± 616.86%ID/g), and small intestine (28.25 ± 33.92%ID/g), indicating partial hepatobiliary clearance. Likewise, elevated levels of [^68^Ga]Ga^III^‐Ent were also observed in bone (4.43 ± 2.01%ID/g) and lungs (11.01 ± 5.07%ID/g), suggesting prolonged retention in the blood. Urine metabolite analysis (Figure S15) showed 13% degradation of the tracer. In contrast, the biodistribution profile of [^68^Ga]Ga^III^‐TREN‐CAM showed favorable pharmacokinetics, with the highest concentration of the radiotracer present in the kidneys (31.94 ± 6.08%ID/g) in agreement with excretion of the tracer via renal routes. Lower concentrations of [^68^Ga]Ga^III^‐TREN‐CAM were detected in the liver (2.01 ± 0.51%ID/g) and small intestine (3.05 ± 0.64%ID/g) indicating reduced hepatobiliary clearance when compared to [^68^Ga]Ga^III^‐Ent. Urine metabolite analysis of [^68^Ga]Ga^III^‐TREN‐CAM showed that only 58% of the tracer remained intact in vivo (Figure S15). Nonetheless, [^68^Ga]Ga^III^‐TREN‐CAM does not significantly accumulate in nontarget tissues, such as bone (1.23 ± 0.07%ID/g), blood (0.66 ± 0.11%ID/g), or heart (0.42 ± 0.10%ID/g), contrasting with the biodistribution profiles of unchelated [^68^Ga]GaCl_3_, which exhibit high uptake in these organs. [49] This demonstrates its high in vivo stability and favorable low‐background biodistribution, highlighting its potential to be further evaluated as an imaging agent for infection. The difference in off‐target tissue uptake is unexpected, with differences in hydrophilicity predicting faster clearance for [^68^Ga]Ga^III^‐Ent. We hypothesize that the mammalian blood protein siderocalin is sequestering the corresponding radiochelates to different extents, resulting in differences in blood retention. Siderocalin is a lipocalin designed to target the bacterial iron acquisition system by sequestering iron‐bound catecholate siderophores such as enterobactin and bacillibactin, among others. [57] While in vitro studies have shown that siderocalin cannot easily distinguish between Ga^III^‐Ent (*K*_d_ = 0.37 nM) and Fe^III^‐Ent (*K*_d_ = 0.41 nM), and that Fe^III^‐TREN‐CAM (*K*_d_ = 0.32 nM) is recognized with comparable affinity, [58] kinetics of binding may be better suited to characterize this interaction in dynamic environments such as the host.

### 2.3 PET/CT Imaging of an *E. Coli* Infection in Mice and Ex Vivo Biodistribution

**FIGURE 6:**
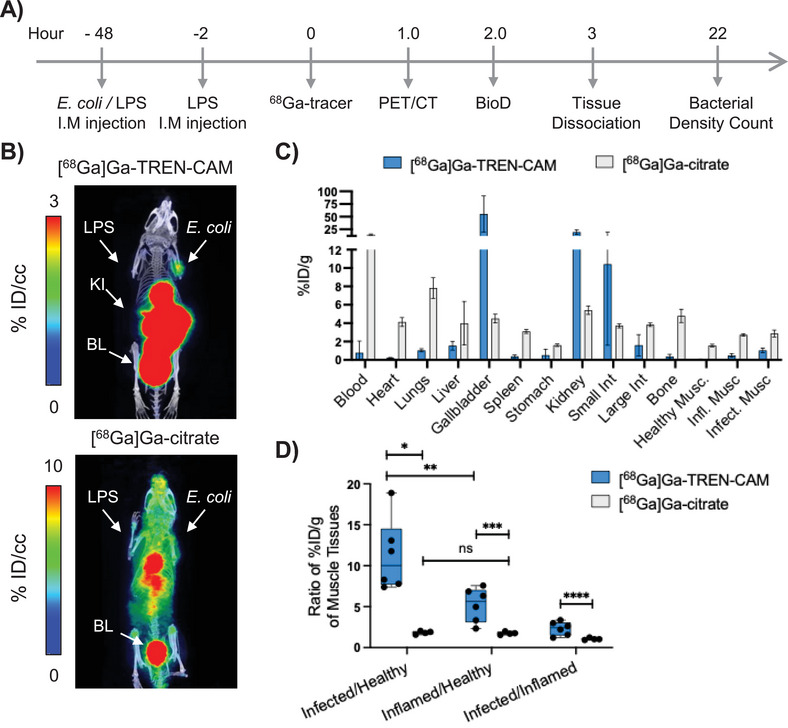
Evaluation of [^68^Ga]Ga^III^‐TREN‐CAM and [^68^Ga]Ga^III^‐citrate in an *E. coli* K12 infection model. (A) Descriptive timeline of the animal study. (B) Representative positron emission tomography/computed tomography images of [^68^Ga]Ga^III^‐TREN‐CAM and [^68^Ga]Ga^III^‐citrate 1 h post administration of radiotracer in mice with an *E.coli* K12 infection. Images are normalized to units of %ID/cc and presented as maximum intensity projection scans (MIPS). Activity is primarily shown in the bladder (BL), kidneys (KI), infected triceps (*E. coli* K12), and inflamed triceps (LPS). % ID/cc is the percentage of injected dose per cubic centimeter. (C) Ex vivo biodistribution profile of [^68^Ga]Ga^III^‐TREN‐CAM and [^68^Ga]Ga^III^‐citrate 2 hours post injection. Error bars represent ±1 standard deviation (*n* = 6 for TREN‐CAM or *n* = 4 for citrate). % ID/g of organ is the percentage of injected dose per gram of organ weight. (D) Muscle tissue‐to‐healthy muscle %ID/g ratio for *E. coli* infected muscle, inflamed muscle, and healthy muscle. Statistical differences were calculated using two‐tailed Student's *t* tests assuming unequal variances; * *p *= 0.0034, ** *p* = 0.0196, *** *p* = 0.0089, **** *p* = 0.0126.

With the selective in vitro uptake of Ga^III^‐TREN‐CAM affirmed, we tested the ability of the radiolabeled analog [^68^Ga]Ga^III^‐TREN‐CAM to serve as a suitable tracer for PET imaging of bacterial infections in vivo. To this end, BALB/c mice (*n* = 6) were inoculated with live *E. coli* K12 (10^6^ CFU) in the right triceps, and lipopolysaccharide (LPS, 27 µg) was injected in the left triceps to provide the contralateral, sterile inflammation control. [39] The infection was allowed to develop for 48 h, upon which the ^68^Ga‐labeled tracer (184–208 µCi for TREN‐CAM or 74–103 µCi for citrate), formulated in DPBS buffer, was administered via tail vein injection (Figure 6a). Following ex vivo biodistribution analysis, microscopic examination of inflamed, infected, and healthy muscle tissue (right thigh) was performed to confirm presence or absence of bacterial colonies (Supporting Information Figures S23 and S24).

PET‐CT images were acquired 1 h post injection (Figures 6b and S21) of the radiotracer, followed by ex vivo biodistribution analysis at 2 h, as illustrated in Figure 6a. The results were compared to those obtained with [^68^Ga]Ga^III^‐citrate (Figures 6b and S22), a ^68^Ga‐based analogue of [^67^Ga]Ga^III^‐citrate which is approved to image infections and inflammation in humans [59, 60].

A strong correlation between PET imaging (Figure 6b,c) and ex vivo biodistribution data was observed. As shown in Figure 6c (tabulated data available in Table S7), elevated levels of [^68^Ga]Ga^III^‐citrate were found in the blood (14.50 ± 1.22%ID/g), as well as highly perfused organs, such as the heart (4.13 ± 0.49%ID/g), lungs (7.83 ± 1.12%ID/g), liver (3.99 ± 2.37%ID/g), and spleen (3.11 ± 0.22%ID/g), in agreement with the high background signal observed in the PET/CT images (Figure 6b). Likewise, high uptake of this tracer was observed in the bone (4.80 ± 0.72%ID/g), consistent with accumulation levels previously documented in healthy BALB/c rodents and established animal models of inflammation. [48, 61] Of note, [^68^Ga]Ga^III^‐citrate shows a tracer uptake in the infected muscle (2.88 ± 0.38%ID/g) significantly higher than the uptake observed in healthy muscle (1.57 ± 0.14%ID/g). Nonetheless, comparable levels are also observed in the inflamed muscle tissue (2.73 ± 0.14%ID/g). Indeed, direct comparison of the inflamed/healthy muscle (1.8 ± 0.2) and infected/healthy muscle (1.7 ± 0.2) ratios (Figure 6d) shows that there is no statistically significant difference, thus highlighting the limitations of this tracer to differentiate bacterial infections from a sterile inflammation.

PET/CT and biodistribution analysis with [^68^Ga]Ga^III^‐TREN‐CAM reveals rapid renal clearance of the tracer (19.54 ± 4.71%ID/g in kidneys), along with low retention in the blood (0.79 ± 1.27%ID/g), liver (1.55 ± 0.47%ID/g), and lungs (1.05 ± 0.17%ID/g). In contrast to [^68^Ga]Ga^III^‐citrate, low heart (0.23 ± 0.05%ID/g) and bone uptake (0.38 ± 0.24%ID/g) were observed, consistent with the PET/CT imaging data (Figure 6b). Notably, [^68^Ga]Ga^III^‐TREN‐CAM showed an infected‐to‐healthy muscle ratio of 11.2 ± 4.4, over two‐fold higher and comparing favorably to the inflamed‐to‐healthy muscle ratio (5.2 ± 2.1) observed for [^68^Ga]Ga^III^‐citrate. These results highlight the ability of [^68^Ga]Ga^III^‐TREN‐CAM to selectively target tissue infected by *E. coli* and perform as a suitable agent capable of differentiating bacterial infections from sterile inflammation. The rapid clearance from nontarget tissues, such as heart, bone, and lung offers opportunities to selectively detect sites of infection in those organs in a diagnostic imaging setting.

Further in vivo studies were conducted to enable a head‐to‐head comparison of [^68^Ga]Ga^III^‐TREN‐CAM to [^68^Ga]Ga^III^‐Ent, a primary endogenous siderophore for *E. coli*. For this study, shorter in vivo incubation times were employed (5 h) due to accelerated bacterial growth observed when utilizing an alternate batch of the *E. coli* K12 strain. Microscopic examination of inflamed, infected, and healthy muscle tissue was also performed to confirm presence or absence of bacterial colonies (Supporting Information Figures S19 and S20).

Our data (Table S6 and Figures S16 and S17) showed a five‐fold increase of [^68^Ga]Ga^III^‐Ent levels in the liver, a two‐fold increase in the bone, and up to a seven‐fold higher localization in nontargeted tissues, such as lungs, as well as blood compared to [^68^Ga]Ga^III^‐TREN‐CAM. No statistically significant differences between [^68^Ga]Ga^III^‐Ent and [^68^Ga]Ga^III^‐TREN‐CAM uptake levels in the infected muscle were observed. This demonstrates the ability of the latter to perform as effectively as the natural siderophore with respect to bacterial uptake within the host. The elevated levels of the tracer in the inflamed muscle compared to healthy muscle in both cases may arise from upregulation of acute phase proteins, such as lipocalins in order to inactivate any harmful pathogens as noted above. [62] Metabolite analysis indicated that approximately 90% of [^68^Ga]Ga^III^‐Ent remained intact in vivo. In contrast, [^68^Ga]Ga^III^‐TREN‐CAM appears to undergo dechelation or chemical modification in vivo, as evidenced by degradation of almost 48% of the tracer as illustrated in Figure S18, observations that are consistent with the metabolite analysis carried out in healthy mice. Despite the apparent degradation of [^68^Ga]Ga^III^‐TREN‐CAM, both ex vivo biodistribution and PET/CT data demonstrate minimal accumulation in off‐target organs.

### 2.4 Treatment Monitoring of an *E. coli* Infection in Mice via PET‐CT Imaging

**FIGURE 7:**
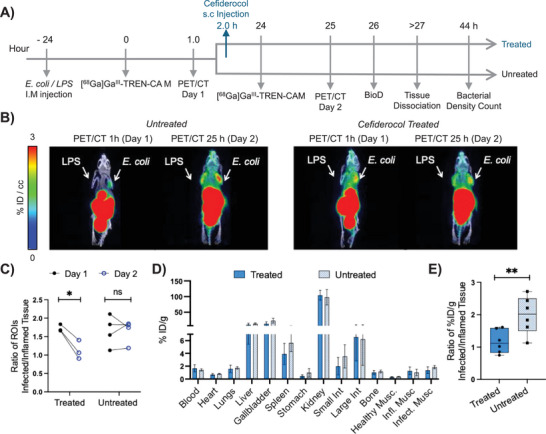
Use of [^68^Ga]Ga^III^‐TREN‐CAM to track the progress of an *E. coli* K12 infection in BALB/c mice upon treatment with cefiderocol. (A) Descriptive timeline of the animal study. (B) Representative positron emission tomography/computed tomography images of [^68^Ga]Ga^III^‐TREN‐CAM showing the progress of the *E. coli* K12 infection in specimens untreated or treated with cefiderocol. Images are collected prior to administration of cefiderocol treatment (or saline control) and 24 h post administration of treatment (or saline vehicle) and are normalized to units of %ID/cc and presented as maximum intensity projection scans (MIPS). Activity is shown in the infected triceps (*E. coli*) and inflamed triceps (LPS). (C) Correlation plot showing the infected muscle‐to‐inflamed muscle ROI ratios for specimens of the treated and untreated cohort before (day 1) and after administration of treatment or saline vehicle (day 2), respectively. Statistical differences were calculated using two‐tailed Student's *t* tests assuming unequal variances; * *p* = 0.0418. Region of interest (ROI) values correspond to the quantified percentage of injected dose per cubic centimeter of organ from the PET/CT images. (D) Ex vivo biodistribution profile of [^68^Ga]Ga^III^‐TREN‐CAM 2 h post injection on day 2 after assessment of antibiotic treatment via PET/CT Imaging. Error bars represent ±1 standard deviation (*n* = 6). % ID/g of organ is the percentage of injected dose per gram of organ weight. (E) Comparison of the infected muscle‐to‐inflamed muscle %ID/g ratios for treated (*n* = 6) and untreated (*n *= 6) cohort before and after treatment. Statistical differences were calculated using two‐tailed Student's *t* tests assuming unequal variances; ** *p*= 0.0181.

Next, we evaluated whether [^68^Ga]Ga^III^‐TREN‐CAM could be used as a tool to monitor the therapeutic response to the siderophore‐based antibiotic cefiderocol in an *E. coli* K12 infection model. To this end, BALB/c mice (*n* = 6 per cohort) were infected with live *E. coli* K12 in the right triceps and received a sterile inflammation control (LPS) in the left triceps. Representative specimens (*n* = 4, unless otherwise stated) per cohort from both untreated and cefiderocol‐treated cohorts underwent PET/CT imaging prior to treatment administration, 25 h post initial inoculation with *E. coli* K12. Mice in the treatment group received a single subcutaneous dose of cefiderocol 26 h post inoculation with the bacterial pathogen, at a dose of ∼40 mg/kg, in accordance with a preclinical treatment protocol for a similar *E. coli* murine infection model published in the literature. [63] The initial PET/CT scans were then compared to images acquired 24 h post administration of antibiotic treatment (49 h after initial inoculation with *E. coli* K12) in order to assess the progression of the infection and the efficacy of the treatment (Figure 7a).

As shown in Figure 7b (coronal PET/CT data for all specimens available in Figures S25 and S26), untreated specimens exhibit a more pronounced increase in tracer uptake (%ID/cc) in infected muscle tissue compared with treated specimens. ROI analysis of the PET/CT images (Tables S8 and S9) was used to quantify the extent of [^68^Ga]Ga^III^‐TREN‐CAM uptake in tissues infected with *E. coli* K12, as well as other major organs and tissues. The infected muscle‐to‐inflamed muscle ROIs ratio (Figure 7c) confirms that, as noted earlier, the cefiderocol‐treated cohort shows a statistically significant decrease from day 1 (1.7 ± 0.097) compared to day 2 (1.1 ± 0.26). In contrast, a significant difference between the muscle‐to‐inflamed muscle ROIs ratios of untreated specimens from day 1 (1.7 ± 0.42) compared to day 2 (1.7 ± 0.31) was not observed.

Following PET/CT imaging post antibiotic treatment, ex vivo biodistribution analysis with [^68^Ga]Ga^III^‐TREN‐CAM was carried out in both untreated and cefiderocol‐treated mice, revealing comparable distribution profiles (Figure 7d and Table S10). Characteristic rapid renal clearance of the tracer was observed, with uptake values of 104.46 ± 15.45%ID/g and 98.07 ± 24.59%ID/g in the kidneys of cefiderocol‐treated and untreated specimens, respectively. Low tracer retention was observed in off‐target tissues of cefiderocol‐treated specimens, including blood (1.67 ± 0.56%ID/g), heart (0.68 ± 0.17%ID/g), and bone (1.02 ± 0.33%ID/g). Comparable levels of tracer accumulation are observed in off‐target organs of untreated mice, with low retention in the blood (1.43 ± 0.19%ID/g), heart (0.80 ± 0.08%ID/g), and bone (1.19 ± 0.22%ID/g). When comparing the infected‐to‐inflamed muscle %ID/g ratios of cefiderocol‐treated and untreated groups (Figure 7e), a reduction in tracer uptake was observed in the treated group (1.17 ± 0.37) relative to the untreated group (1.99 ± 0.58). This observation can be attributed to the reduced bacterial burden in the antibiotic‐treated cohort, resulting from the antibacterial activity of cefiderocol against *E. coli*. This was further supported by ex vivo microbiology analysis, which confirmed a decrease in bacterial density in the infected triceps muscle of the treated cohort (5.44 ± 3.75 10^7^ CFU/g) compared to the untreated cohort (1.03 ± 0.98 10^8^ CFU/g) (Figures S27–S32 and Table S11).

## 3 Conclusion

TREN‐CAM is a tripodal tris‐catecholate chelator that mimics the structure of Enterobactin, a siderophore and virulence factor utilized by *E. coli*, and members of the Enterobacteriaceae family, as well as other pathogenic bacteria causative of common infectious diseases. We have investigated the use of ^67/68^Ga‐radiolabeled TREN‐CAM as an adequate probe for the selective imaging of bacterial cells in vitro and in vivo. In vitro studies indicated that [^67^Ga]Ga^III^‐TREN‐CAM, readily mimics [^67^Ga]Ga^III^‐Ent with respect to uptake and accumulation: it associates with *E. coli K12, S. aureus* RN4220, and *P. aeruginosa* PAO1. In comparison with the hydroxamate‐based siderophore, [^67^Ga]Ga^III^‐DFO, which accumulates efficiently in the Gram‐positive organism *S. aureus* RN4220, [^67^Ga]Ga^III^‐TREN‐CAM shows enhanced uptake in Gram‐negative organisms *E. coli* K12 and *P. aeruginosa* PAO1 in direct correlation with the growth inhibitory activity of the siderophore‐antibiotic cefiderocol. The direct comparison with [^67^Ga]Ga^III^‐Ent and blocking studies suggests selective uptake via the FepA, as well as the Cir/Fiu transporter system.

In vivo studies in naïve rodents revealed that both [^68^Ga]Ga^III^‐TREN‐CAM and [^68^Ga]Ga^III^‐Ent exhibit rapid renal clearance and similar distribution profiles. However, [^68^Ga]Ga^III^‐Ent showed elevated blood residency and off‐target accumulation. In vivo studies in an *E. coli* K12 infection model demonstrated that [^68^Ga]Ga^III^‐TREN‐CAM displays a favorable biodistribution with high in vivo stability and low blood retention. Although both tracers demonstrate comparable accumulation in the infected muscle tissue, [^68^Ga]Ga^III^‐Ent exhibits increased nonspecific uptake in off‐target organs, including lungs, bone, heart, and liver, as well as the muscle treated with LPS (inflammation control) and the healthy muscle tissue (non‐treated). Similarly, direct comparison of [^68^Ga]Ga^III^‐TREN‐CAM and the clinically utilized tracer [^68^Ga]Ga^III^‐citrate showed that the latter exhibits comparable uptake in inflamed and infected muscle tissue, thereby limiting its ability to distinguish aseptic inflammation from bacterial infection. In contrast, [^68^Ga]Ga^III^‐TREN‐CAM shows a favorable uptake profile, enabling differentiation between inflammation and infection based on in vivo PET/CT imaging and ex vivo biodistribution studies.

Additionally, the ability of [^68^Ga]Ga^III^‐TREN‐CAM to function not only as a diagnostic tool for bacterial infections but also to assess the therapeutic response to the siderophore‐based antibiotic cefiderocol was evaluated in an *E. coli* K12 infection model. Both ex vivo biodistribution studies and PET/CT imaging revealed a good correlation between bacterial burden and the levels of radiotracer in infected tissues, enabling the assessment of antibiotic treatment efficacy noninvasively.

Overall, our findings demonstrate that TREN‐CAM performs well as an enterobactin mimic, with its ^67/68^Ga‐complex showing promising in vitro results as a selective probe for Gram‐negative bacteria susceptible to treatment with the catechol‐antibiotic cefiderocol. This work also shows that when paired with cefiderocol, [^68^Ga]Ga^III^‐TREN‐CAM serves as an example of a theranostic pair for the treatment of infection: [^68^Ga]Ga^III^‐TREN‐CAM selectively visualizes *E. coli* soft tissue bacterial infections through ^68^Ga PET imaging with low residual radioactivity in bone, heart, and lung tissue. This is especially relevant as those organs are frequently involved in life‐threatening clinical infections (endocarditis, prosthetic joint infections, and pneumonia) that could benefit from diagnostic imaging to streamline treatment. In addition to established antibiotics, we highlight the potential of ^68^Ga‐TREN‐CAM to be employed in tandem with a clinically employed catechol‐based siderophore‐antibiotic, further expanding the application of the theranostic paradigm for infectious disease applications.

## Conflicts of Interest

The authors declare no conflicts of interest.

## Supporting information




**Supporting File**: Radiosynthesis of tracers, additional characterization data, microbiology and animal protocols are summarized in the Supporting Information. The authors have cited additional references within the Supporting Information [1–7].

## Data Availability

The data that support the findings of this study are available on request from the corresponding author. The data are not publicly available due to privacy or ethical restrictions.
