# Supplementary material for: Positron Emission Tomography Imaging of Bacterial Infections With an Enterobactin Analog to Monitor Treatment Efficacy With a Catechol Antibiotic
Source: Angew Chem Int Ed Engl. 2026 Mar 11;65(17):e2391261. doi: 10.1002/anie.2391261 (PMC13098312; doi:10.1002/anie.2391261)
Supplement: Supplementary file 1 — Supporting File: Radiosynthesis of tracers, additional characterization data, microbiology and animal protocols are summarized in the Supporting Information. The authors have cited additional references within the Supporting Information [1–7]. [file ANIE-65-e2391261-s001.pdf]

Supporting Information for:

Positron Emission Tomography Imaging of Bacterial Infection with an Enterobactin Analog to Monitor Treatment Efficacy with a Catechol Antibiotic

M. Andrey Joaqui-Joaqui,[a,b]‡ Phuong Nguyen Tran,[a] ‡ Axia Marlin,[a] Fiona Armstrong-Pavlik,[b] Minhua Cao,[a] Valérie C. Pierre*[b,c], and Eszter Boros,*[a]

a) Department of Chemistry, University of Wisconsin Madison, 1101 University Avenue, Madison, Wisconsin, 53705, USA

b) Department of Chemistry, University of Minnesota, Minneapolis, 207 Pleasant St SE, Minneapolis, MN 55455, United States

c) Department of Chemistry, University of Utah, 315 South 1400 East, Room 2020, Salt Lake City, UT 84112, United States

Table of Contents

1 General Considerations....4

1.1 Spectroscopy and mass spectrometry methods: 4

1.2 High Performance Liquid Chromatography Methods:....4

1.2.1 Analytical purification method 4

1.2.2 Analysis of ^{67}Ga-labeled compounds and non-radioactive references 4

1.2.3 Analysis of ^{68}Ga-labeled compounds and non-radioactive references. 5

2 Chemical synthesis of ligands and non-radioactive complexes 5

2.1 Synthesis of Fe^{III}-Ent....5

2.2 Synthesis of Ga^{III}-Ent 6

3 Synthesis of Radiochemical Complexes....9

3.1 Radiosynthesis of ^{67}Ga-labeled Tracers 9

3.1.1 Preparation of [^{67}Ga]$GaCl_3$ 9

3.1.2 Radiosynthesis of [^{67}Ga]Ga^{III}-DFO....9

3.1.3 Radiosynthesis of [^{67}Ga]Ga^{III}-TREN-CAM....9

3.2 Radiosynthesis of ^{68}Ga-labeled Tracers 10

3.2.1 Radiosynthesis of [^{68}Ga]Ga^{III}-TREN-CAM....10

3.2.2 Radiosynthesis of [^{68}Ga]Ga^{III}-Ent 11

4 In vitro Bacterial Assays....11

4.1 Bacterial Cell Uptake in different bacterial strains with non-radioactive tracers 12

4.2 Bacterial Cell Uptake in different bacterial strains with ^{67}Ga-labeled tracers 13

4.3 Cell Uptake Competition Assay in *E. coli* K12 14

4.4 Antimicrobial Activity Assay....14

5 In vivo performance assessment of ^{68}Ga-labeled tracers....15

5.1 Formulations of [^{68}Ga]Ga^{III}-TREN-CAM and [^{68}Ga]Ga^{III}-Ent in DPBS 15

5.2 In vivo evaluation of [^{68}Ga]Ga^{III}-TREN-CAM and [^{68}Ga]Ga^{III}-Ent in naïve mice 15
5.2.1 Ex vivo biodistribution in naïve mice models: 15
5.2.2 Urine metabolite analysis....................16
5.3 In vivo evaluation of [^{68}Ga]Ga^{III}-TREN-CAM and [^{68}Ga]Ga^{III}-Ent in E. coli infected mice 17
5.3.1 Bacteria inoculum preparation, infection animal model, and administration of ^{68}Ga-radiotracer 17
5.3.2 PET-CT imaging....................17
5.3.3 Ex vivo biodistribution 18
5.3.4 Urine metabolite analysis....................19
5.3.5 Tissue collection and cell culture....................20
5.3.6 Histopathology....................21
5.4 Comparative in vivo evaluation of [^{68}Ga]Ga^{III}-TREN-CAM and [^{68}Ga]Ga^{III}-citrate in *E. coli* infected mice....................23
5.4.1 Bacteria inoculum preparation, infection animal model, and administration of ^{68}Ga-radiotracer 23
5.4.2 PET-CT imaging and ex vivo biodistribution....................23
5.4.3 Tissue collection and cell culture....................25
5.5 Assessment of Cefiderocol treatment in an *E. coli* K12 infected mice via PET-CT using [^{68}Ga]Ga^{III}-TREN-CAM as radiotracer 26
5.5.1 PET/CT imaging, Antibiotic Treatment, ROI analysis....................26
5.5.2 Ex vivo biodistribution 29
5.5.3 Tissue collection and bacterial density assessment 31

Abbreviations

Ac	Acetate
aq	aqueous
CPM	Counts per Minute
CPS	Counts per Second
DMSO	Dimethyl sulfoxide
DPBS	Dulbecco's Phosphate-Buffered Saline
Ent	Enterobactin
ESI	Electrospray Ionization
HEPES	4-(2-hydroxyethyl)-1-piperazineethanesulfonic acid
HPLC	High Performance Liquid Chromatography
HR	High Resolution
ICP-OES	Inductively Coupled Plasma- Optical Emission Spectroscopy
LPS	Lipopolysaccharide
MIPS	Maximum Intensity Projection Scan
MS	Mass Spectrometry
NaOAc	Sodium acetate
NH_4OAc	Ammonium acetate
NMR	Nuclear Magnetic Resonance
PET/CT	Positron Emission Tomography / Computed Tomography
ROI	Region of Interest
s.c	Subcutaneous
TRIS	2-hydroxymethylpropane-1,3-diol

1 General Considerations

Unless otherwise stated, all starting materials were purchased from commercial sources and used without further purification.

1.1 Spectroscopy and mass spectrometry methods:

Nuclear Magnetic Resonance (NMR) Spectroscopy: All NMR data was collected on a Bruker Advance III 400 spectrometer at the University of Minnesota, and a Bruker Avance-500 spectrometer at the University of Wisconsin-Madison Department of Chemistry Paul Bender Chemical Instrumentation Center (CIC). Chemical shifts are reported as parts per million (ppm) and are referenced relative to TMS or residual solvent peaks. Deuterated solvents were obtained from Cambridge Isotope Laboratories (Tewskbury, MA, USA).

Mass spectrometry: Mass spectrometry data was collected on a Thermo Scientific Q Exactive Focus Orbitrap MS system at the University of Wisconsin-Madison Department of Chemistry Paul Bender Chemical Instrumentation Center (CIC).

Ultraviolet-Visible (UV-Vis) spectra: UV−visible spectra were recorded on a NanoDrop 1C instrument (AZY1706045) with 1 cm quartz cuvettes.

1.2 High Performance Liquid Chromatography Methods:

1.2.1 Analytical purification method

Purification and analytical HPLC analysis of metal complexes was carried out using an Agilent 1260 Infinity II system equipped with a binary gradient pump, an autosampler, and a UV-detector set to detect UV absorption at 220 nm and 254 nm.

Method A: binary solvent system (A: 10 mM ammonium formate pH 7.4; B: MeCN); gradient (0–2 min: 5% B; 2–14 min: 5–95% B; 14–16 min: 95% B; 16–16.5 min: 95–5% B; 16.5–20 min 5% B); flow rate: 0.8 mL/min; Phenomenex Luna C18 column (5 µm, 150 mm × 3 mm, 100 Å, AXIA packed).

1.2.2 Analysis of ^{67}Ga-labeled compounds and non-radioactive references

^{67}Ga-radiolabeled complexes and non-radioactive references were analyzed on an Agilent 1260 Infinity II system equipped with a binary gradient pump, an autosampler, and a UV-detector set to detect UV absorption at 220 nm and 254 nm. The instrument was coupled to an Eckert & Ziegler photomultiplier tube detector (Type B-FC-3600) with 2” lead shielding to enable RadioHPLC analyses.

Method B: A = 10 mM NaOAc pH 4.5, B = CH_3CN. Gradient: 0-2 min: 5% B; 2-16 min: 5-95%B; 16-18 min: 95%B; 18-19 min: 95-5%B; 19-20 min: 5%B. Flow rate: 0.8 mL/min. Column: Phenomenex Luna® 5 µm C18(2) column (100 Å, 150 × 3 mm).

1.2.3 Analysis of ^{68}Ga-labeled compounds and non-radioactive references.

^{68}Ga-radiolabeled complexes and non-radioactive references were analyzed on an Agilent 1260 Infinity II system equipped with a binary gradient pump, an autosampler, and a UV-detector set to detect UV absorption at 220 nm and 254 nm. The instrument was coupled to a LabLogic 1" NaI photomultiplier tubedetector with 2" lead shielding to enable RadioHPLC analyses.

Method C: A = 10 mM NaOAc pH 4.5, B = CH_3CN. Gradient: 0-2 min: 5% B; 2-16 min: 5-95%B; 16-18 min: 95%B; 18-19 min: 95-5%B; 19-20 min: 5%B. Flow rate: 0.8 mL/min. Column: Phenomenex Luna® 5 µm C18(2) column (100 Å, 150 × 3 mm).

2 Chemical synthesis of ligands and non-radioactive complexes

The non-radioactive Ga^{3+} complexes of TREN-CAM and DFO were synthesized as previously reported by our group with successful synthesis determined by ^{1}H NMR, RP-HPLC and HR-ESI-MS.[1-2] Enterobactin, on the other hand, was purchased from Sigma Aldrich and used without any additional purification.

2.1 Synthesis of Fe^{III}-Ent

Fe^{III}-enterobactin (Fe^{III}-Ent) was synthesized adapting a protocol previously reported by Dertz.[3] Briefly, Fe^{III}-enterobactin was formed overnight at room temperature in 20% DMSO in H_2O in the presence of a 1:1 ratio ligand: $FeCl_3$ (concentration determined by ICP-OES). After 16 h at room temperature, the solvent was removed under reduced pressure and the resulting solid was redissolved in MeOH and purified via RP-HPLC (Method A) to yield Fe^{III}-Ent as a purple solid (yield: 2.0 mg, 95 %).

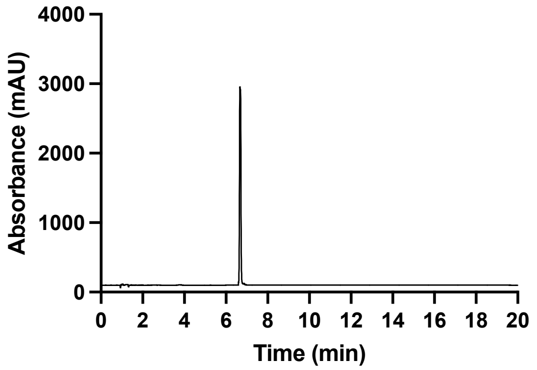

Figure S1. Chromatographic analysis of Fe^{III}-Ent. Absorbance was monitored at 254 nm.

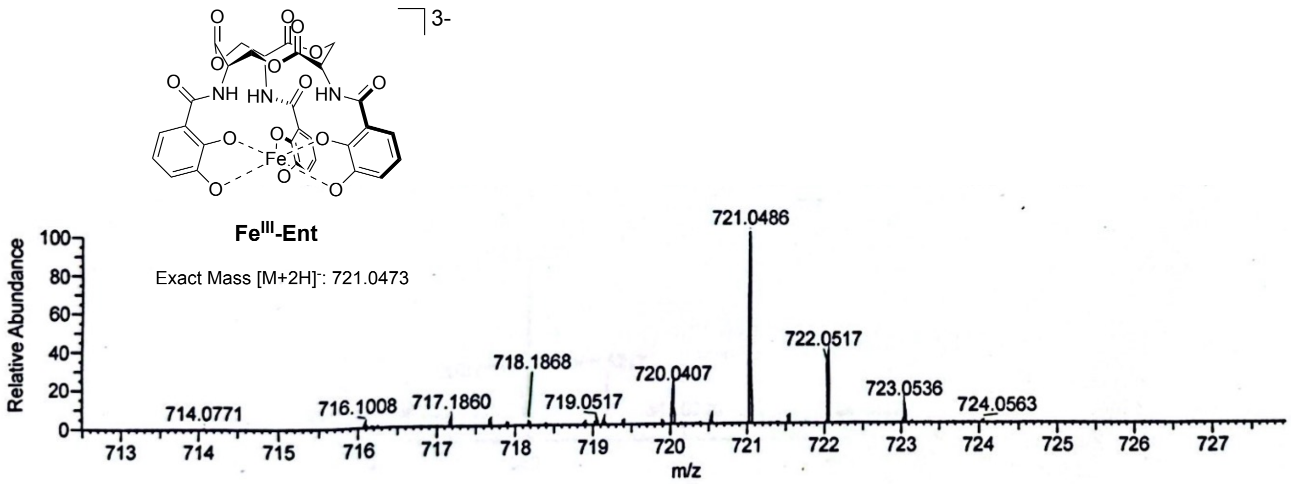

Figure S2. High-resolution ESI(-) mass spectrum of Fe^{III}-Ent.

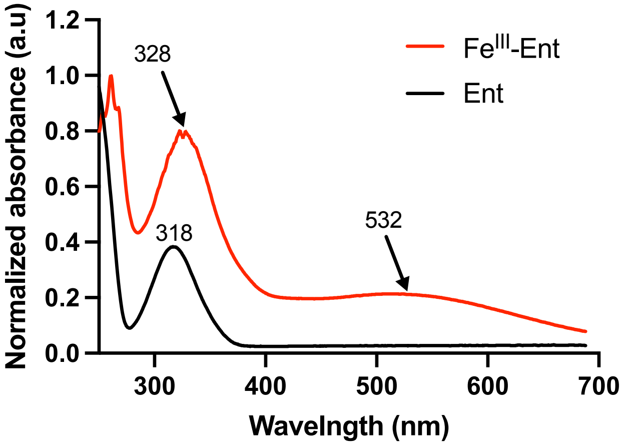

Figure S3. Comparative optical absorption spectra of Enterobactin (Ent) and its corresponding Fe^{III} metal complex (Fe^{III}-Ent) in water.

2.2 Synthesis of Ga^{III}-Ent

Ga^{III}-enterobactin (Ga^{III}-Ent) was formed overnight at room temperature in MeOH in the presence of a 1:1 ratio ligand:Ga(acac)$_3$ (concentration determined by ICP-OES). The resulting product was purified via RP-HPLC (Method A) to yield Ga^{III}-Ent as a white solid (yield: 3.0 mg, 93 %).

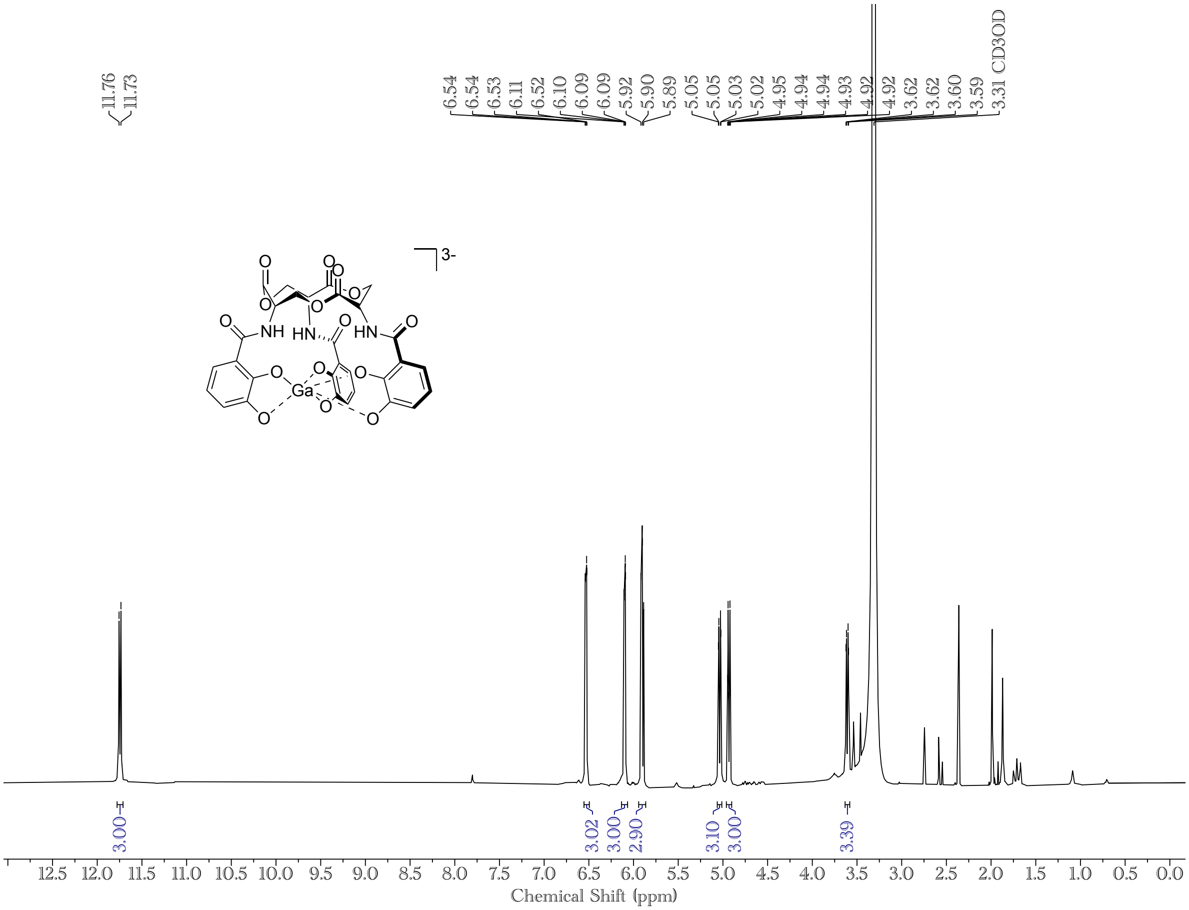

Figure S4. ^{1}H NMR of Ga^{III}-Ent, 500 MHz, CD_3OD (crude NMR). The impurities around 2.0-2.5 ppm are attributed to the presence of the acetylacetonate ligand from the $Ga(acac)_3$ salt used.

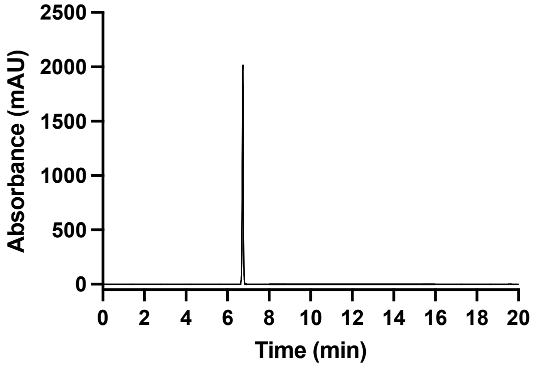

Figure S5. Chromatographic analysis of Ga^{III}-Ent. Absorbance was monitored at 254 nm**.**

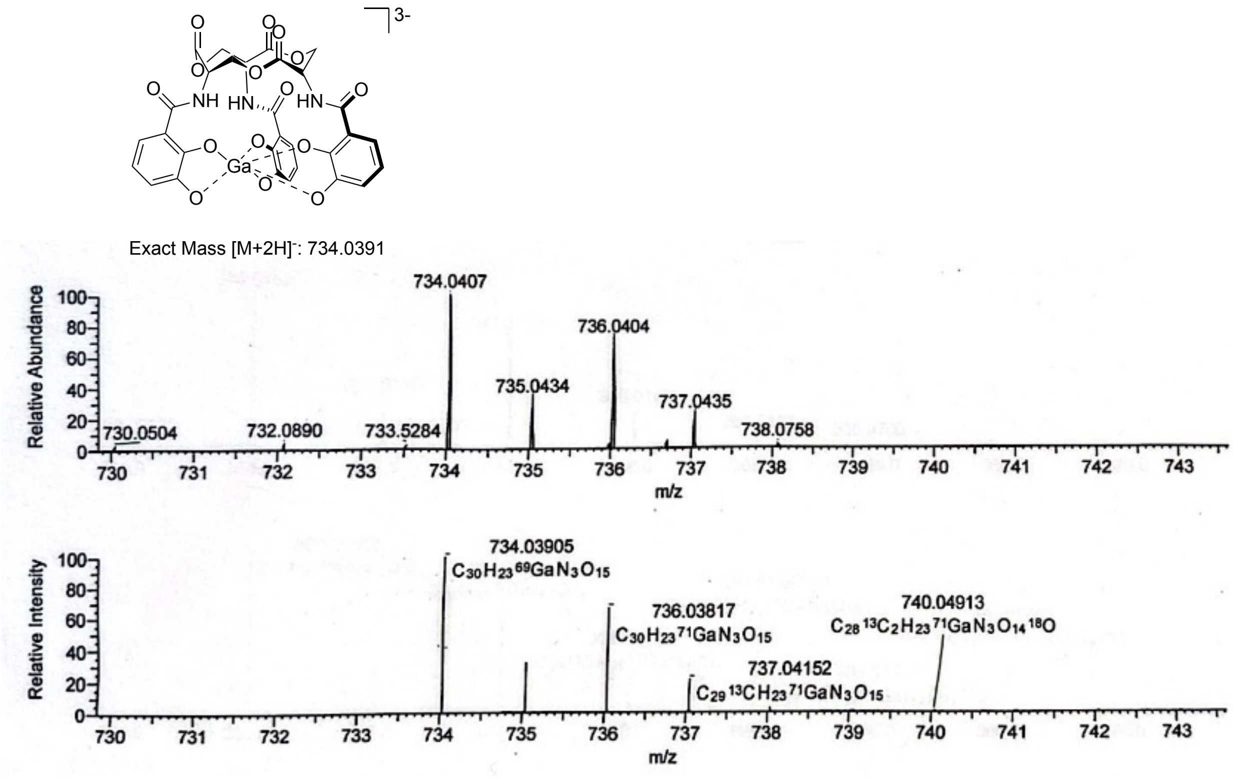

Figure S6. Experimental (top) and calculated (bottom) High-resolution ESI(-) mass spectrum of Ga^{III}-Ent.

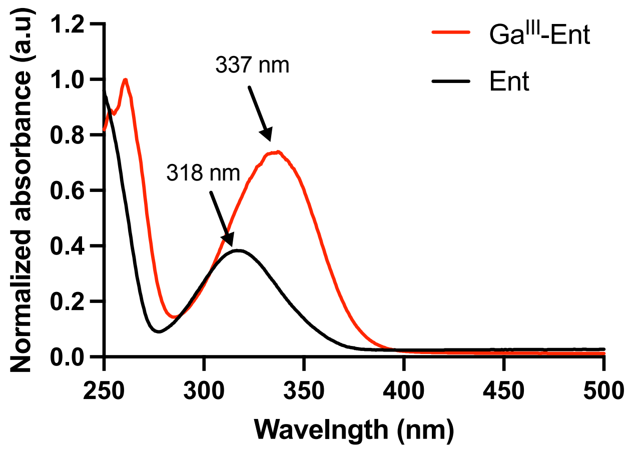

Figure S7. Comparative optical absorption spectra of Enterobactin (Ent) and its corresponding Ga^{III} metal complex (Ga^{III}-Ent) in water.

3 Synthesis of Radiochemical Complexes

3.1 Radiosynthesis of ^{67}Ga-labeled Tracers

3.1.1 Preparation of [^{67}Ga]GaCl$_3$

[^{67}Ga]Ga^{III}-citrate was purchased from Jubilant Radiopharma and received at an average specific activity of 1.5 mCi/mL. This [^{67}Ga]Ga^{III}-citrate solution was converted to [^{67}Ga]GaCl$_3$ using a previously described protocol.[4-5]

3.1.2 Radiosynthesis of [^{67}Ga]Ga^{III}-DFO

[^{67}Ga]GaCl$_3$ (26 µL, 85 µCi) was added to a solution of DFO (30 µL, 0.4 mM) in water. The pH of the labeling solution was adjusted to 7 using HEPES buffer (150 µL, 50 mM, pH 7.4). Quantitative labeling was confirmed via radio-HPLC (Method B, R_t = 4.63 min – Absorbance monitored at 220 nm).

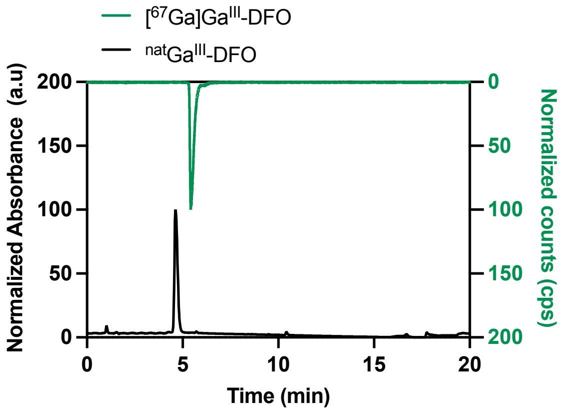

Figure S8. Chromatographic analysis of ^{67}Ga-labeled DFO and its corresponding non-radioactive complex. Absorbance of non-radioactive samples was monitored at 220 nm.

3.1.3 Radiosynthesis of [^{67}Ga]Ga^{III}-TREN-CAM

[^{67}Ga]GaCl$_3$ (28 µL, 90 µCi) was added to a solution of TREN-CAM (30 µL, 0.4 mM) in DMSO/H_2O (20/80). The pH of the labeling solution was adjusted to 7 using NH_4OAc (150 µL, 50 mM, pH 8.8). Quantitative labeling was confirmed via radio-HPLC (Method B, R_t = 6.79 min – Absorbance monitored at 254 nm).

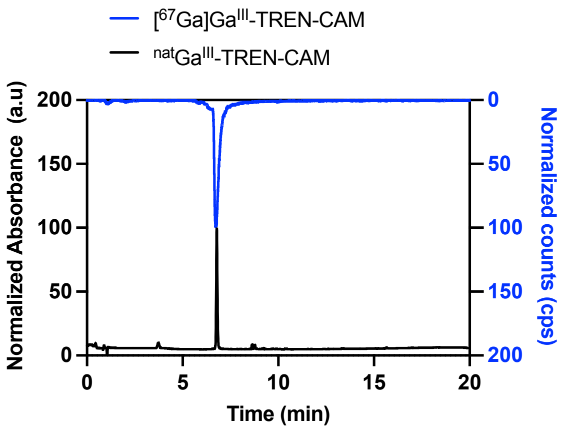

Figure S9. Chromatographic analysis of ^{67}Ga-labeled TREN-CAM and its corresponding non-radioactive complex. Absorbance of non-radioactive samples was monitored at 254 nm

3.2 Radiosynthesis of ^{68}Ga-labeled Tracers

[^{68}Ga]$GaCl_3$ was obtained from the Radiopharmaceutical Production Facility (RPF) located within the Wisconsin Institutes of Medical Research, at the University of Wisconsin School of Medicine and Public Health. This material was received as an eluate of [^{68}Ga]$GaCl_3$ in 0.1 M HCl at an average specific activity of 15-18 mCi/mL.

[^{68}Ga]Ga-citrate was synthesized from [^{68}Ga]$GaCl_3$ as previously described in the literature.[6]

3.2.1 Radiosynthesis of [^{68}Ga]Ga^{III}-TREN-CAM

[^{68}Ga]$GaCl_3$ (110 µL, 1.485 mCi) was added to a solution of TREN-CAM (45 µL, 1 mM) in DMSO/H_2O (20/80). The pH of the labeling solution was adjusted to 7 using NH_4OAc (50 µL, 1 M, pH 8). Quantitative labeling was confirmed via radio-HPLC (Method C, R_t = 6.85 min – Absorbance monitored at 254 nm).

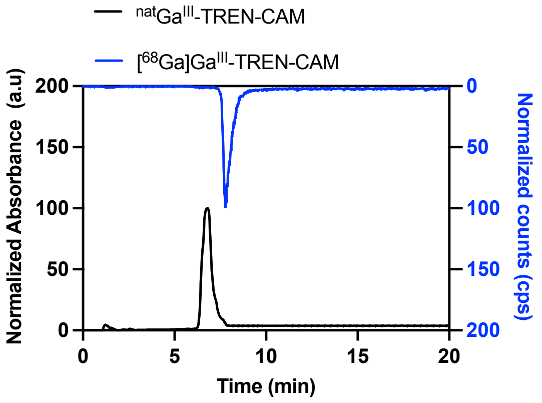

Figure S10. Chromatographic analysis of ^{68}Ga-labeled TREN-CAM and its corresponding non-radioactive complex. Absorbance of non-radioactive samples was monitored at 254 nm.

3.2.2 Radiosynthesis of [^{68}Ga]Ga^{III}-Ent

[^{68}Ga]$GaCl_3$ (64 µL, 1.223 mCi) was added to a solution of enterobactin (100 µL, 0.4 mM) in DMSO/H_2O (20/80). The pH of the labeling solution was adjusted to 7 using HEPES (100 µL, 0.5 M, pH 8.8). Quantitative labeling was confirmed via radio-HPLC (method C, R_t = 6.30 min – Absorbance monitored at 254 nm).

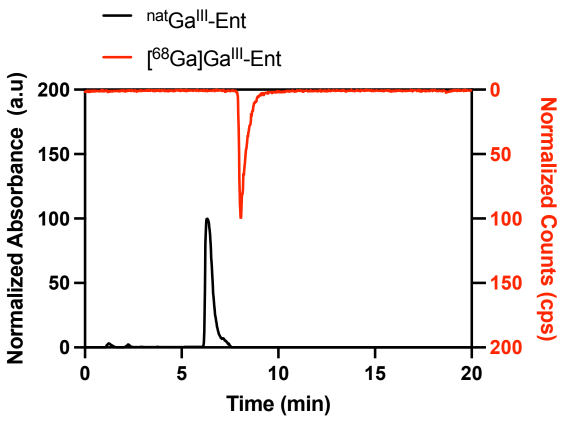

Figure S11. Chromatographic analysis of ^{68}Ga-labeled enterobactin and its corresponding non-radioactive complex. Absorbance of non-radioactive samples was monitored at 254 nm.

4 In vitro Bacterial Assays

Luria-Bertani (LB) broth media (tryptone 10 g/L, yeast extract 5 g/L, NaCl 10 g/L) was purchased from Fisher Scientific and prepared in purified water according to the manufacturer's instructions. The resulting solution was autoclaved to ensure sterility. To make the broth iron deficient, 4.06 mL of a 1 mg/mL sterile (autoclaved) solution of 2,2′-bipyridine (DP) was added to 250 mL of LB media.

Mueller–Hinton broth (MHB) media was purchased from Fisher Scientific and prepared according to the company instructions. In order to make the broth Ca^{2+}/Mg^{2+} adjusted as well as iron-deficient, 4.06 mL of 1 mg/mL sterile (autoclaved) aq. solution of 2,2′-bipyridine (DP), 418 µL of sterile 1 M $CaCl_2$ and 155 µL of sterile 1 M $MgCl_2$ were added to 250 mL of sterile MHB broth.

T-media was prepared by dissolving the contents mentioned in Table S1 in 0.8 L of water, followed by addition of 12 M HCl to adjust the pH to 7.4. The final volume was adjusted to 1 L by adding water as necessary.

Table S1. Biological media, inorganic salts and aminoacids used for the preparation of T-media solution.

Compound	Amount	Compound	Amount
NaCl	6.0714 g	L-Leucine	58.2 mg
KCl	3.7034 g	L-Proline	60.0 mg
$CaCl_2$	141.3 mg	L-Tryptophan	51.0 mg
$MgCl_2 \cdot 6H_2O$	112.1 mg	Thiamine hydrochloride	7.5 mg
NH_4Cl	122.5 mg	Glucose	2.0128 g
TRIS	12.0313 g	Bacto™ Casa aminoacids	55.1 mg
Na_2SO_4	157.2 mg	KH_2PO_4	32.7 mg

4.1 Bacterial Cell Uptake in different bacterial strains with non-radioactive tracers

Association of $^{nat}Ga^{III}$-TREN-CAM and $^{nat}Ga^{III}$-DFO with bacteria was investigated with *Klebsiella pneumoniae* ATCC 10031, *Acinetobacter baumannii*, *Pseudomonas aeruginosa* ATCC 25619, *Enterobacter cloacae*, and *Escherichia coli* K12. Briefly, bacteria were aerobically grown overnight LB medium at 37 °C. After 18 hours, bacteria (200 µL from the overnight culture were inoculated into 50 mL of T-media and incubated at 37 °C for 6 hours. After this time, 9 mL of this culture (10^9 CFU/mL) were mixed with 1 mL of the $^{nat}Ga^{III}$-TREN-CAM or $^{nat}Ga^{III}$-DFO stock solution (100 µM in H_2O) and incubated for 10 min at 37°C. Bacteria was pelleted by centrifuging the cell culture at 10,000 rpm. The resulting pellets were washed with PBS buffer (3 × 1 mL). Recovered cell pellets were then re-suspended in BPER lysis buffer and divided into two aliquots: one for ICP analysis (25%) and one for BCA protein content analysis (75%). The samples for ICP were digested with 5% HNO_3 overnight at 120 °C in sealed ampoules. After digestion, each sample was diluted with deionized water to reach a final concentration of 1% HNO_3. The concentration of Ga in each sample was determined by ICP-MS analysis (ALS Environmental, Salt Lake City, UT). Bacterial protein content was separately determined using a Micro BCA™ Protein Assay Kit (Thermo Scientific). Each experiment was repeated six times (n=6) from three independent bacterial cultures. The time and concentration dependence of association of $^{nat}Ga^{III}$-TREN-CAM and $^{nat}Ga^{III}$-DFO with *E. coli* K12 was also assessed as a function of incubation time (10 and 30 min) and concentration of the Ga(III) complex (1 and 10 µM).

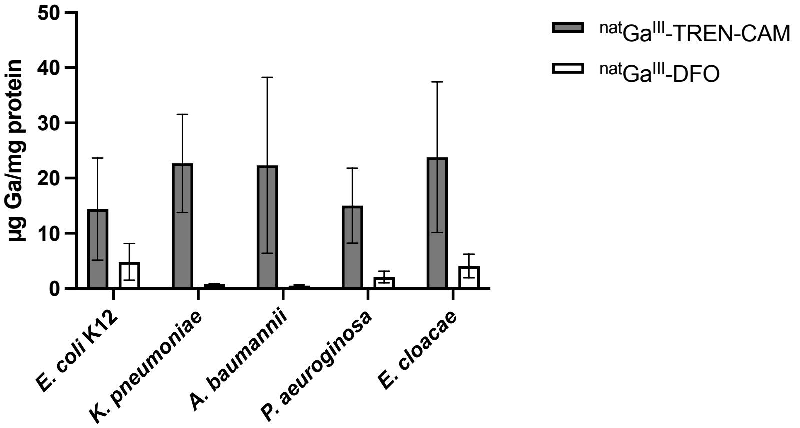

Figure S12. Uptake of $^{nat}Ga^{III}$-TREN-CAM and $^{nat}Ga^{III}$-DFO by different bacterial strains.

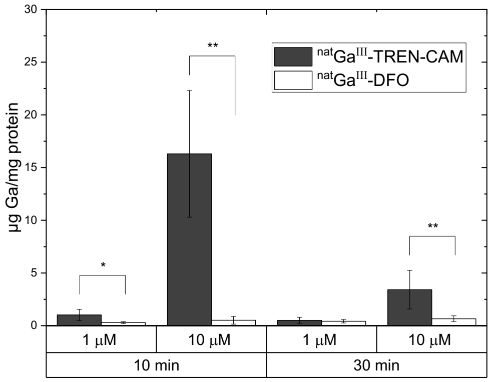

Figure S13. Uptake of $^{nat}Ga^{III}$-TREN-CAM and $^{nat}Ga^{III}$-DFO in *E. coli* K12 at different concentrations and times of incubation. Error bars represent standard deviations (n = 3). ∗ indicates a statistically significant difference (two samples t test; (∗) $p < 0.05$, (∗∗) $p < 0.03$).

4.2 Bacterial Cell Uptake in different bacterial strains with ^{67}Ga-labeled tracers

E. coli K12, *S. aureus* RN4220 and *P. aeruginosa* PO1 were incubated overnight in 10 mL of LB iron-deficient medium at 37°C (210 rpm). The overnight cultures were then diluted 1:10 in 10 mL of LB iron-deficient medium and incubated at 37°C until an OD_{600} of 0.4 was reached (1 h for both *E. coli* K12 and *P. aeruginosa* PO1, and 45 min for *S. aureus* RN4220). Uptake was initiated by adding [^{67}Ga]Ga^{III}-DFO (23 µL, 10 µCi) or [^{67}Ga]Ga^{III}-TREN-CAM (22 µL, 10 µCi) to falcon tubes containing 10 mL bacterial culture and incubating at 37°C in an incubator shaker. Aliquots (1 mL) were removed after 10 min, 20 min, 30 min, 60 min, and 120 min and centrifuged for 3 min (14500 rpm). The supernatant was removed, and the pellet was washed with DPBS (3 x 500 µL). The assay was performed in five replicates. The amount of remaining radioactivity in the bacterial cell pellet was estimated via automated Liquid Scintillation Counter (PerkinElmer, Tri-Carb 2900TR).

Uptake was expressed as the % decay-corrected radioactivity found in the pellet:

$$\% \text{ uptake} = \frac{\text{cpm (pellet)}}{\text{cpm reference } (10\ \mu Ci)}$$

Table S2. Percentage of internalized [^{67}Ga]Ga^{III}-DFO and [^{67}Ga]Ga^{III}-TREN-CAM in *E. coli* K12 upon incubation in iron-deficient medium (n = 5 or n = 3).

Incubation time (min)	[^{67}Ga]Ga^{III}-DFO (%)	[^{67}Ga]Ga^{III}-TREN-CAM (%)
10	0.20 ± 0.02	2.55 ± 0.21
20	0.24 ± 0.05	5.67 ± 0.33
30	0.35 ± 0.11	8.40 ± 0.94
60	0.80 ± 0.28	11.03± 3.21
120	2.47 ± 2.02	8.33 ± 1.83

Table S3. Percentage of internalized [^{67}Ga]Ga^{III}-DFO and [^{67}Ga]Ga^{III}-TREN-CAM in *P. aeruginosa* PAO1 upon incubation in iron-deficient medium (n = 5 or n = 3).

Incubation time (min)	[^{67}Ga]Ga^{III}-DFO (%)	[^{67}Ga]Ga^{III}-TREN-CAM (%)
10	0.47 ± 0.07	4.17 ± 0.67
20	0.55 ± 0.10	4.08 ± 0.62
30	0.67 ± 0.16	3.79 ± 1.25
60	0.94 ± 0.24	4.08 ± 0.31
120	2.23 ± 0.22	9.60 ± 1.47

Table S4. Percentage of internalized [^{67}Ga]Ga^{III}-DFO and [^{67}Ga]Ga^{III}-TREN-CAM in *S. aureus* RN4220 upon incubation in iron-deficient medium (n = 5).

Incubation time (h)	[^{67}Ga]Ga^{III}-DFO (%)	[^{67}Ga]Ga^{III}-TREN-CAM (%)
10	40.25 ± 2.91	1.33 ± 0.90
20	75.37 ± 4.88	1.50 ± 1.00
30	95.15 ± 10.39	1.80 ± 1.15
60	106.55 ± 4.24	2.40 ± 1.18
120	112.98 ± 7.09	4.69 ± 1.58

4.3 Cell Uptake Competition Assay in *E. coli* K12

E. coli K12 was incubated overnight in 10 mL of LB iron-deficient medium at 37°C (210 rpm). The overnight cultures were then diluted 1:10 in 10 mL of LB iron-deficient medium and incubated at 37°C until an OD_{600} of 0.4 was reached. Bacteria were incubated with 200x excess of Fe^{III}-Ent to ligand concentration 2 h prior incubation with the corresponding [^{67}Ga]Ga^{III}-Ent and [^{67}Ga]Ga^{III}-TREN-CAM. Uptake was carried out following the protocol in section 4.1

4.4 Antimicrobial Activity Assay

The antibacterial activity of Cefiderocol against *E. coli* K12, *S. aureus* RN4220 and *P. aeruginosa* PO1 was evaluated by estimating its minimum inhibitory concentration (MIC) using the microdilution method according to the Clinical and Laboratory Standards Institute (CLSI) guidelines. MIC experiments were conducted in triplicates and carried out at 3 different instances, resulting in n = 9 for each reported MIC_{98} to ascertain a maximum error margin of <5% for each reported MIC values. Briefly, bacteria were incubated overnight in 5 mL of MHB iron-deficient medium at 37°C (210 rpm). The overnight bacterial cultures were then diluted in 1:100 in 5 mL of MHB iron-deficient medium and incubated until OD_{600} = 0.6 was reached (3 h for all strains). Daytime cultures were diluted in 1:500 in 50 mL of MHB iron-deficient broth prior to inoculating the 96-well polypropylene treatment plate.

A stock solution of Cefiderocol was prepared in 20% DMSO in H_2O and the concentration determined via UV-Vis using the molar extinction coefficient reported in the literature.[7]

10 µL solution of Cefiderocol (0.3 mM) was added to the first well of the 96-well plate and serial dilutions were made down each row of the plate. 40 µL of growth media and 50 µL of diluted bacterial inoculum was also added to each well, resulting in a total volume of 100 µL and a concentration gradient of 0.3 × 10^{-4} M to 0.92 × 10^{-12} M. The plates were incubated at 37 °C for 18 h and each plate was examined for bacterial growth using a plate reader (Biotek Synergy HTX multi-mode plate reader). The MIC_{98} was recorded as the lowest compound concentration (µM) required to inhibit >90% of bacterial growth as judged by the absorbance of the culture media relative to the negative control.

5 In vivo performance assessment of ^{68}Ga-labeled tracers

Female, Balb/c, mice were purchased from The Jackson Laboratory. All animal experiments were conducted with the approval of the University of Wisconsin-Madison Institutional Animal Care and Use Committee (IACUC). All studies were conducted in accordance with the relevant guidelines and regulations and approved under protocol number M006738 (PI: Boros) and conducted at UW-Madison School of Medicine and Public Health, at the Small Animal Imaging and Radiotherapy Facility (SAIRF).

5.1 Formulations of [^{68}Ga]Ga^{III}-TREN-CAM and [^{68}Ga]Ga^{III}-Ent in DPBS

Aliquots of radiolabeled [^{68}Ga]Ga^{III}-TREN-CAM (7.2 µCi/µL in NH_4OAc buffer pH 7) or [^{68}Ga]Ga^{III}-Ent (5.7 µCi/µL in HEPES buffer pH 7) were diluted with sterile DPBS 1X in order to reach a final radioactive concentration of 1.0-1.8 µCi/µL. The resulting PBS formulation was vortexed followed by quality analysis via radio-HPLC before injections into rodents.

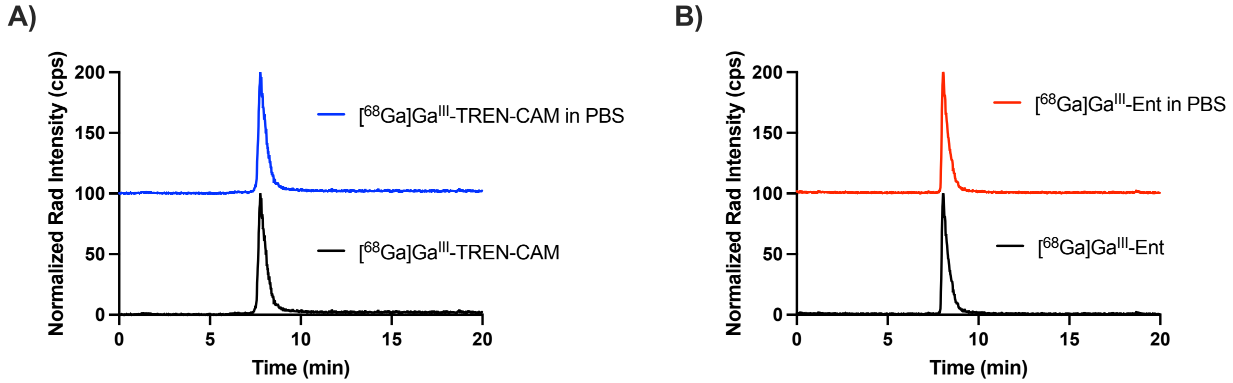

Figure S14. Comparative chromatographic analysis of the initially labeled ^{68}Ga-labeled radiotracers and their corresponding PBS formulations. A) [^{68}Ga]Ga^{III}-TREN-CAM. B) [^{68}Ga]Ga^{III}-Ent.

Freshly prepared [^{68}Ga]Ga^{III}-citrate was diluted in DPBS 1X so that the final concentration of sodium citrate was 80 mM before proceeding to inject into rodents.

5.2 In vivo evaluation of [^{68}Ga]Ga^{III}-TREN-CAM and [^{68}Ga]Ga^{III}-Ent in naïve mice

5.2.1 Ex vivo biodistribution in naïve mice models:

Biodistribution profiles of [^{68}Ga]Ga^{III}-TREN-CAM and [^{68}Ga]Ga^{III}-Ent were first evaluated in healthy naïve mice. To this end, 100 µL (containing 100-150 µCi of radiotracer) of [^{68}Ga]Ga^{III}-TREN-CAM or [^{68}Ga]Ga^{III}-Ent formulated in PBS 1X buffer were administered via tail vein injection into 6 week old female balb/c mice (n = 4 per cohort). Mice were euthanized 1.5 hours post-injection and biodistribution studies were conducted. Urine and blood were collected, organs were harvested, intestinal content removed, and activity assessed via a gamma counting (Hidex Automatic Gamma Counter). Recorded measurements of counts per minute (cpm) were decay-corrected to the time of injection of the ^{68}Ga-radiotracer, converted to units of Ci by using the efficiency of the Gamma Counter for Ga-68, and the amount of activity present in each organ was expressed as percentage of injected dose per gram of organ weight (% ID/g).

Table S5. Tabulated values for the biodistribution in healthy mice of [^{68}Ga]Ga^{III}-TREN-CAM and **[**[^{68}Ga]Ga^{III}-Ent (n = 4) at 1.5 hours post-injection. Values expressed as % ID/g. Error expressed as ± 1 SD for n = 4.

**Organs**	**[^{68}Ga]Ga^{III}-TREN-CAM**	**[^{68}Ga]Ga^{III}-Ent**	**P Value**
Blood	0.66 ± 0.11	2.14 ± 0.99	0.05638
Heart	0.42 ± 0.10	1.43 ± 0.91	0.11296
Lung	4.49 ± 0.81	11.01 ± 5.07	0.08060
Liver	2.01 ± 0.51	10.99 ± 5.15	0.03915
Gallbladder	140.04 ± 113.49	745.71 ± 616.86	0.14313
Spleen	1.01 ± 0.27	2.59 ± 1.08	0.05642
Pancreas	0.54 ± 0.09	1.93 ± 1.39	0.13949
Kidney	31.94 ± 6.08	90.53 ± 26.55	0.01883
Stomach	0.95 ± 0.48	2.25 ± 2.27	0.34024
Small intestine	3.05 ± 0.64	28.25 ± 33.92	0.23402
Muscle	0.49 ± 0.11	1.74 ± 1.08	0.10197
Bone	1.23 ± 0.07	4.43 ± 2.01	0.05026
Tail	5.54 ± 2.11	25.26 ± 14.48	0.07080

5.2.2 Urine metabolite analysis

Metabolite analysis was performed by analyzing aliquots of 100 µL of mouse urine collected during biodistribution studies. If the collected urine volume was less than 100 µL, 1X DPBS was added in order to bring it up to a final volume of 100 µL. In cases where the radioactivity present in urine decayed to levels too low for detection by HPLC, fractions eluted from the radioHPLC were collected every 30 seconds, and the amount of radioactivity was estimated via gamma counting (Hidex Automatic Gamma Counter). The chromatogram was then reconstructed by plotting the recorded counts per minute recorded for each fraction as a function of time.

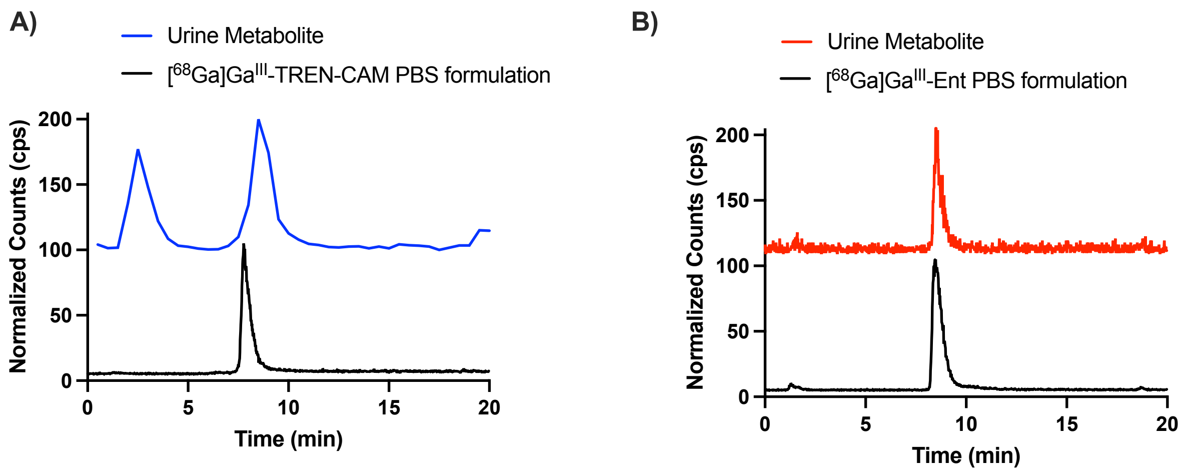

Figure S15. Comparative chromatography analysis of the initial radiotracer PBS formulation and the metabolite analysis of urine collected at 1.5 hours post injection of the radiotracer. A) Reconstructed plot for [^{68}Ga]Ga^{III}-TREN-CAM. B) [^{68}Ga]Ga^{III}-Ent.

5.3 In vivo evaluation of [^{68}Ga]Ga^{III}-TREN-CAM and [^{68}Ga]Ga^{III}-Ent in E. coli infected mice

5.3.1 Bacteria inoculum preparation, infection animal model, and administration of ^{68}Ga-radiotracer

E. coli K12 was inoculated into LB iron-deficient medium (10 mL) and incubated in a shaking incubator at 37°C and 210 rpm overnight. The overnight cultures were then diluted 1:10 in fresh LB iron-deficient medium and incubated at 37°C (210 rpm) until the OD_{600} = 0.3 - 0.4 (45 min) was reached corresponding to a concentration of 1.2 - 1.6 x 10^8 CFU/mL. Six weeks old female balb/c mice were anesthetized with isoflurane and a 60 µL aliquot of bacterial inoculum (1.2 - 1.6 x 10^8 CFU/mL) was injected in the right triceps of the mouse (infected muscle), followed by administration of LPS (30 µL of LPS at 0.9 mg/mL) in the left triceps to provide a contralateral sterile inflammation control (inflamed muscle).

The microbial infection was allowed to develop for 5 hours. Upon development of infection/inflammation, 100 µL (containing 100-180 µCi of radiotracer) of the PBS formulation of [^{68}Ga]Ga^{III}-TREN-CAM or [^{68}Ga]Ga^{III}-Ent was administered into mice (n = 4 per cohort) via tail vein injection.

5.3.2 PET-CT imaging

To determine the pharmacokinetics of the imaging agent, PET-CT scans were collected 1 hour post injection of the radiotracer. The two mice of each cohort that received the highest doses (180 uCi) were used for this purpose. Scans were acquired in a Siemens Inveon Hybrid MicroPET/CT Scanner (Siemens Medical Solutions USA, Inc., Knoxville, TN). Mice were initially anesthetized isoflurane gas and maintained during scans at 2% isoflurane in oxygen. CT scans were acquired prior to PET scans for anatomical coregistration as well as attenuation correction. CT scan parameters were as follows: 220 rotation degrees, 120 rotation steps, binning factor of 4, exposure time of 250 ms, x-ray energy of 80 kVp, 1 mA current, and 105 µm resolution. PET scans were acquired with 40 million coincidence events per mouse, an energy window of 350-650 keV, and a timing window of 3.432 ns. PET-CT images were

normalized to units of percentage of injected dose per cubic centimeter and presented as maximum intensity projected scan (MIPS) images.

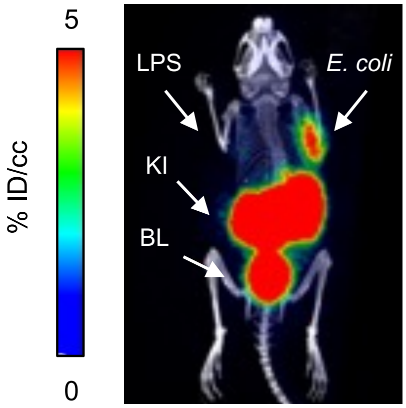

Figure S16. Representative PET/CT image of [^{68}Ga]Ga^{III}-TREN-CAM 1 hour post administration of radiotracer in a mouse with an *E. coli* K12 infection. Image normalized to units of %ID/g and presented as maximum intensity projection scans (MIPS). Activity is primarily shown in the bladder (BL), kidneys (KI), infected triceps (*E. coli*) and inflamed triceps (LPS).

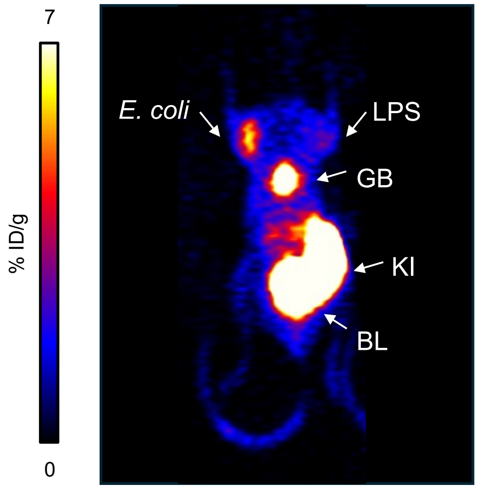

Figure S17. Representative positron emission tomography image of [^{68}Ga]Ga^{III}-Ent 1 hour post administration of radiotracer in a mouse with an *E. coli* K12 infection. Images are normalized to units of %ID/g and presented as maximum intensity projection scans (MIPS). Activity is primarily shown in the bladder (BL), kidneys (KI), gallbladder (GB), infected triceps (*E. coli*) and inflamed triceps (LPS).

5.3.3 Ex vivo biodistribution

Following PET-CT imaging, mice (n= 4 per cohort) were euthanized 1.5 hours post-injection of radiotracer. Urine and blood were collected, organs were harvested, and activity assessed via a gamma counting (Hidex Automatic Gamma Counter). Recorded measurement of counts per minute (cpm) were decay-corrected to the time of injection of the ^{68}Ga-radiotracer, converted to microcurie (μCi) units by using the efficiency of

the Gamma Counter for Ga-68, and the amount of activity present in each organ was expressed as percentage of injected dose per gram of organ weight (% ID/g).

Table S6. Tabulated values for the biodistribution in *E. coli* K12 infected mice of $[^{68}Ga]Ga^{III}$-TREN-CAM and $[^{68}Ga]Ga^{III}$-Ent (n = 4) at 1.5 hours post-injection of radiotracer. Values expressed as % ID/g. Error expressed as ± 1 SD for n = 4.

**Organs**	**$[^{68}Ga]Ga^{III}$-TREN-CAM**	**$[^{68}Ga]Ga^{III}$-Ent**	**P value**
Blood	2.02 ± 1.21	14.00 ± 3.60	0.00431
Heart	0.72 ± 0.14	3.41 ± 0.89	0.00826
Lung	2.09 ± 0.44	15.71 ± 5.80	0.01796
Liver	2.98 ± 0.36	14.23 ± 2.43	0.00230
Spleen	0.91 ± 0.06	4.59 ± 0.64	0.00135
Kidney	315.29 ± 38.93	3.97 ± 0.83	0.00053
Small Intestine	2.07 ± 0.45	2.70 ± 1.61	0.50132
Bone	1.84 ± 0.95	3.84 ± 1.56	0.09010
Infected Muscle	6.47 ± 1.51	7.23 ± 1.51	0.49920
Inflamed Muscle	2.17 ± 0.53	4.92 ± 0.44	0.00024
Healthy Muscle	0.60 ± 0.14	1.21 ± 0.26	0.00166
Tail	3.53 ± 1.34	17.42 ± 10.94	0.00372

5.3.4 Urine metabolite analysis

Metabolite analysis was performed as previously described for healthy mice in section 5.2.2.

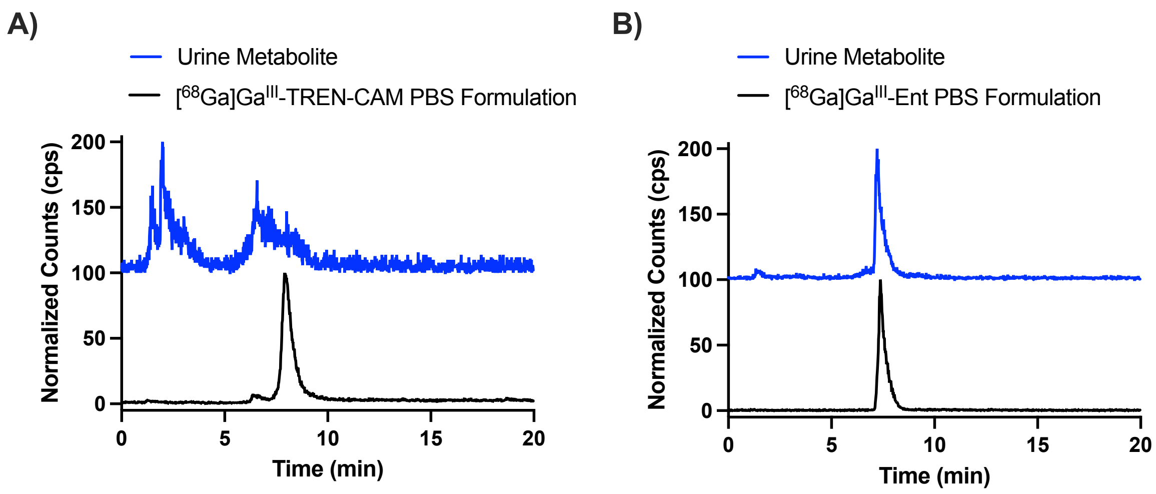

Figure S18. Comparative chromatography analysis of the initial radiotracer PBS formulation and the metabolite analysis of urine collected from *E. coli* K12 infected mice, at 1.5 hours post injection of the radiotracer. A) $[^{68}Ga]Ga^{III}$-TREN-CAM. B) $[^{68}Ga]Ga^{III}$-Ent.

5.3.5 Tissue collection and cell culture

Muscle tissues from the infected right triceps, inflamed left triceps, and healthy quadriceps were collected immediately after biodistribution studies to confirm the presence of viable *E. coli* K12 cells at the infection site and their absence in the healthy and inflamed tissues. The harvested samples were placed in gentleMACS™ C tubes, combined with 5 mL of sterile 1X DPBS buffer, and homogenized using a gentleMACS™ Dissociator for 40 minutes. The resulting homogenates were plated on LB-agar plates and incubated overnight at 37 °C.

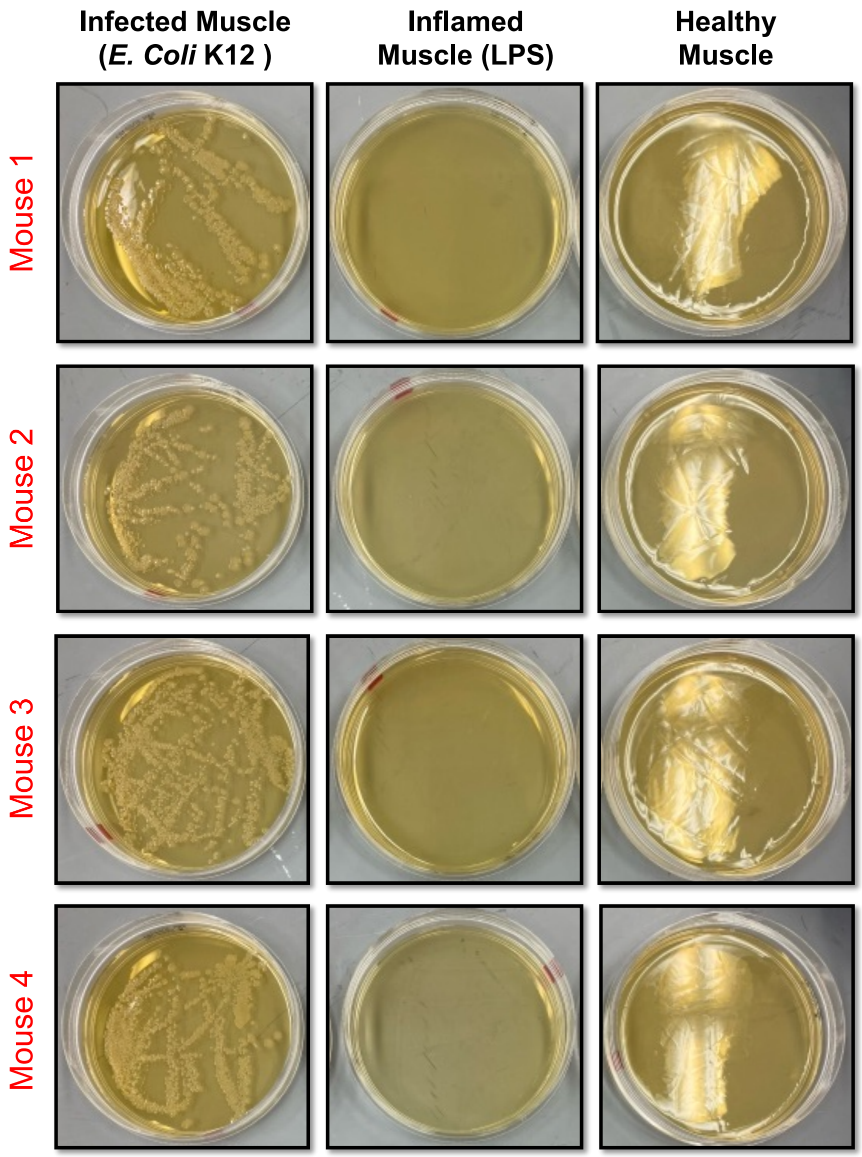

Figure S19. Growth of bacterial colonies on LB-agar plates inoculated with the homogenization products derived from *E. coli* K12 infected muscle tissue, inflamed muscle tissue (LPS induced), and healthy muscle tissue. Viable bacterial colonies are only observed in the plates inoculated with the *E. coli* K12 infected sample tissues.

5.3.6 Histopathology

Infected (right triceps) and inflamed (left triceps) tissues were fixed in 10% formalin solution neutral buffered overnight at 4 °C. Fixed tissues were suspended in 70% ethanol and submitted to the Translational Research Initiatives in Pathology (TRIP) laboratory located in the Department of Pathology and Laboratory Medicine at the University of Wisconsin – Madison, for embedding, and sectioning (5 µm) onto slides for Gram, and Hematoxylin and Eosin (H&E) staining. Stained slides were imaged using an Aperio AT2 Digital pathology slide scanner system and processed using the Leica Aperio Image Scope software version 12.3.3.

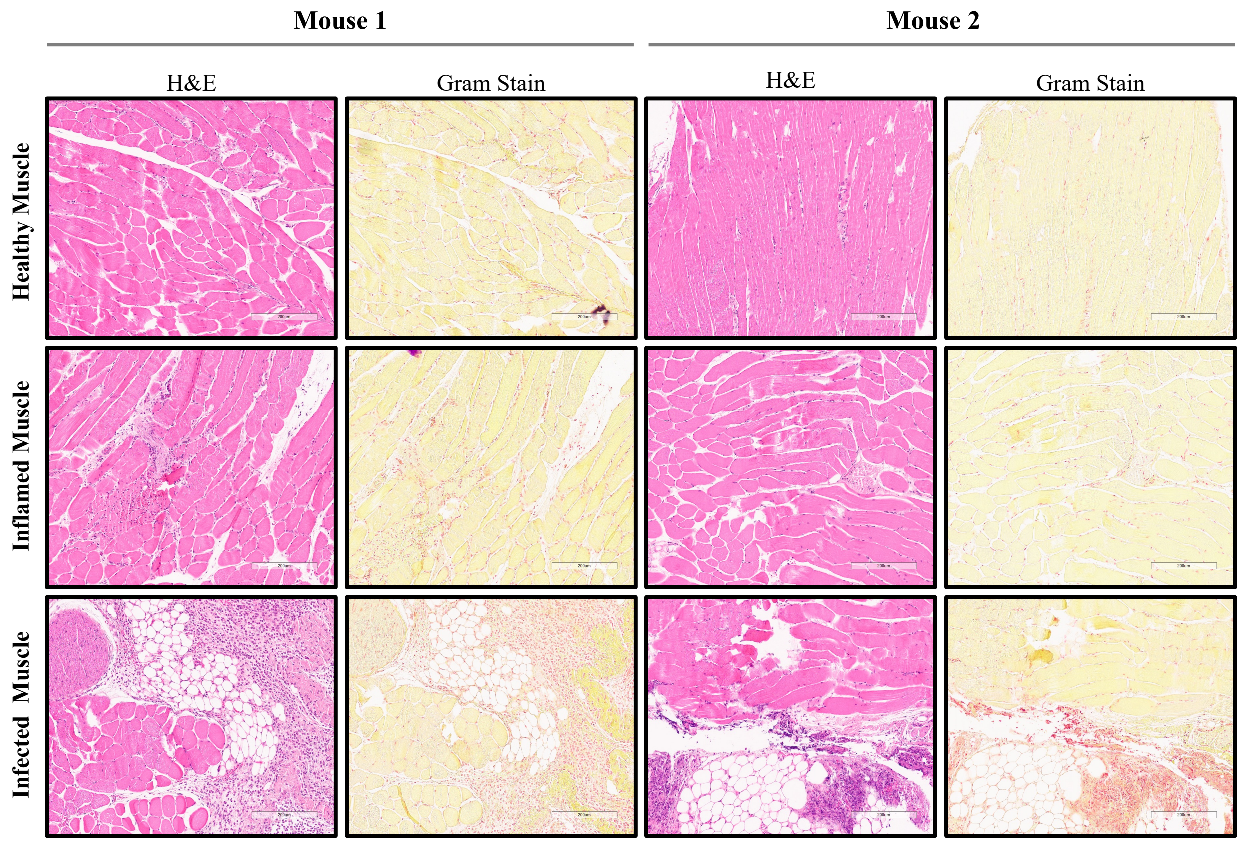

Figure S20. Representative histological images of healthy, sterile-inflamed, and *E. coli* K12 infected muscle tissue slices stained with Gram and H&E. Scale bar, 200 µm.

5.4 Comparative in vivo evaluation of [^{68}Ga]Ga^{III}-TREN-CAM and [^{68}Ga]Ga^{III}-citrate in *E. coli* infected mice

5.4.1 Bacteria inoculum preparation, infection animal model, and administration of ^{68}Ga-radiotracer

E. coli K12 bacterial inoculum was prepared as described in section 5.3.1. Next, six weeks old female balb/c mice were anesthetized with isoflurane and a 60 µL aliquot of bacterial inoculum (1.2 - 1.6 x 10^8 CFU/mL) was injected in the right triceps of the mouse (infected muscle), followed by administration of LPS (30 µL of LPS at 0.9 mg/mL) in the left triceps to provide a contralateral sterile inflammation control (inflamed muscle). The bacterial infection was allowed to develop for 48 hours followed by administration of 100-150 µL of the PBS formulation of [^{68}Ga]Ga^{III}-TREN-CAM (184 – 208 µCi) or [^{68}Ga]Ga^{III}-citrate (74 – 103 µCi) via tail vein injections.

5.4.2 PET-CT imaging and ex vivo biodistribution

PET-CT scans were collected 1-hour post-injection of the radiotracer (49 hours post inoculation with *E. coli* K12). Four mice of each cohort were selected and used for this purpose and scanned simultaneously. Scans were acquired in a Mediso nanoScan® PET/CT Scanner. Mice were initially anesthetized with isoflurane gas scans at 2% isoflurane in oxygen and maintained during scans at 1.5%. PET-CT images were normalized to units of percentage of injected dose per cubic centimeter and presented as maximum intensity projected scan (MIPS) images.

Following PET-CT imaging, mice (n= 6 for [^{68}Ga]Ga^{III}-TREN-CAM or n=4 for [^{68}Ga]Ga^{III}-citrate) were euthanized 2 hours post-injection of radiotracer (50 hours post inoculation with *E. coli* K12) and biodistribution analysis conducted as previously described in section 5.3.3.

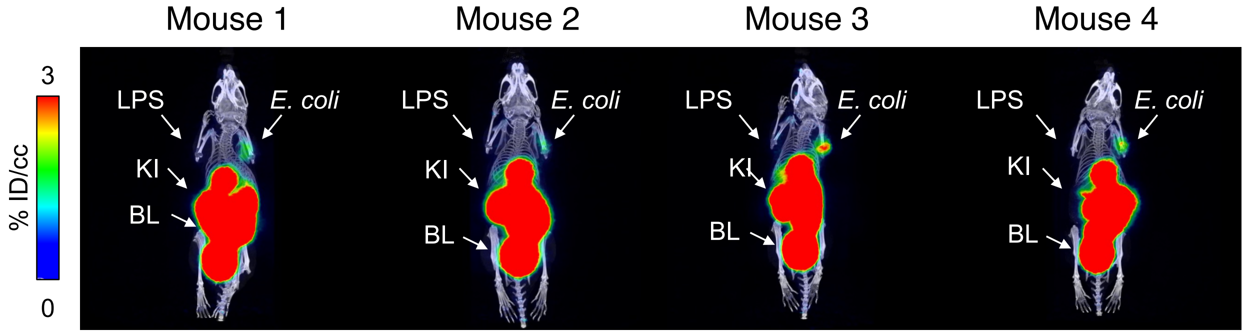

Figure S21. PET/CT images of [^{68}Ga]Ga^{III}-TREN-CAM in BALB/c mice bearing an *E. coli* K12 infection. Images were collected 1 h postinjection of radiotracer (50 hours post inoculation with *E. coli* K12**)** and are normalized to units of %ID/cc and presented as maximum intensity projection scans (MIPS). Arrows point to organs of major interest: infected muscle (*E. coli*), inflamed muscle (LPS), Kidneys (Ki), and bladder (BL).. The % ID/cc is the percentage of injected dose per cubic centimeter.

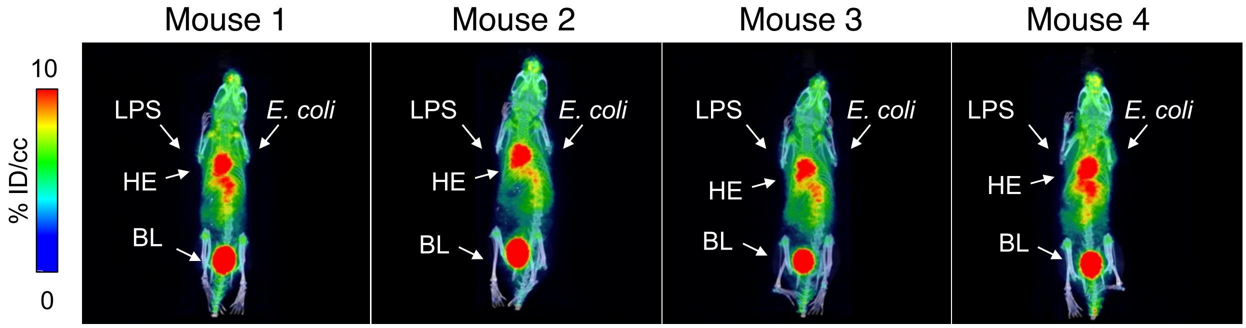

Figure S22. PET/CT images of [^{68}Ga]Ga^{III}-citrate in BALB/c mice bearing an *E. coli* K12 infection. Images were collected 1 h postinjection of radiotracer (50 hours post inoculation with *E. coli* K12) and are normalized to units of %ID/cc and presented as maximum intensity projection scans (MIPS). Arrows point to organs of major interest: infected muscle (E. coli), inflamed muscle (LPS), Heart (HE), and bladder (BL). The % ID/cc is the percentage of injected dose per cubic centimeter.

Table S7. Tabulated values for the biodistribution in *E. coli* K12 infected mice of [^{68}Ga]Ga^{III}-TREN-CAM (n=6) and [^{68}Ga]Ga^{III}-citrate (n=4) at 2 hours post-injection of radiotracer. Values expressed as % ID/g. Error expressed as ± 1 SD.

Organs	[^{68}Ga]Ga^{III}-TREN-CAM	[^{68}Ga]Ga^{III}-citrate	P value
Blood	0.79 ± 1.27	14.50 ± 1.22	< 0.00001
Heart	0.23 ± 0.05	4.13 ± 0.49	0.000499
Lungs	1.05 ± 0.17	7.83 ± 1.12	0.001081
Liver	1.55 ± 0.47	3.99 ± 2.37	0.129164
Gallbladder	55.36 ± 35.54	4.51 ± 0.48	0.017200
Spleen	0.39 ± 0.15	3.11 ± 0.22	0.000005
Stomach	0.53 ± 0.64	1.60 ± 0.13	0.008338
Kidney	19.54 ± 4.71	5.41 ± 0.46	0.000675
Small Intestine	10.42 ± 8.82	3.71 ± 0.21	0.121525
Large Intestine	1.61 ± 1.15	3.83 ± 0.19	0.011724
Bone	0.38 ± 0.24	4.80 ± 0.72	0.000651
Infected Muscle	1.05 ±0.25	2.88 ± 0.38	0.000500
Inflamed Muscle	0.50 ± 0.19	2.73 ± 0.14	< 0.00001
Healthy Muscle	0.10 ±0.02	1.57 ± 0.14	0.000235

5.4.3 Tissue collection and cell culture

Muscle tissues from the infected right triceps, inflamed left triceps, and healthy quadriceps were collected and dissociated as described in section 5.3.5.

Ten microliters of each resulting homogenate were ten-fold serial diluted in sterile DPBS 1X. Diluted solutions (10 µL) were spotted on LB-agar medium (5 dilutions per agar plate). The plate was then tilted 90° to allow the homogenate aliquot run down the surface without cross-contaminating adjacent lanes. The plate was then incubated overnight (16 hours) at 37 °C for CFU enumeration the next day.

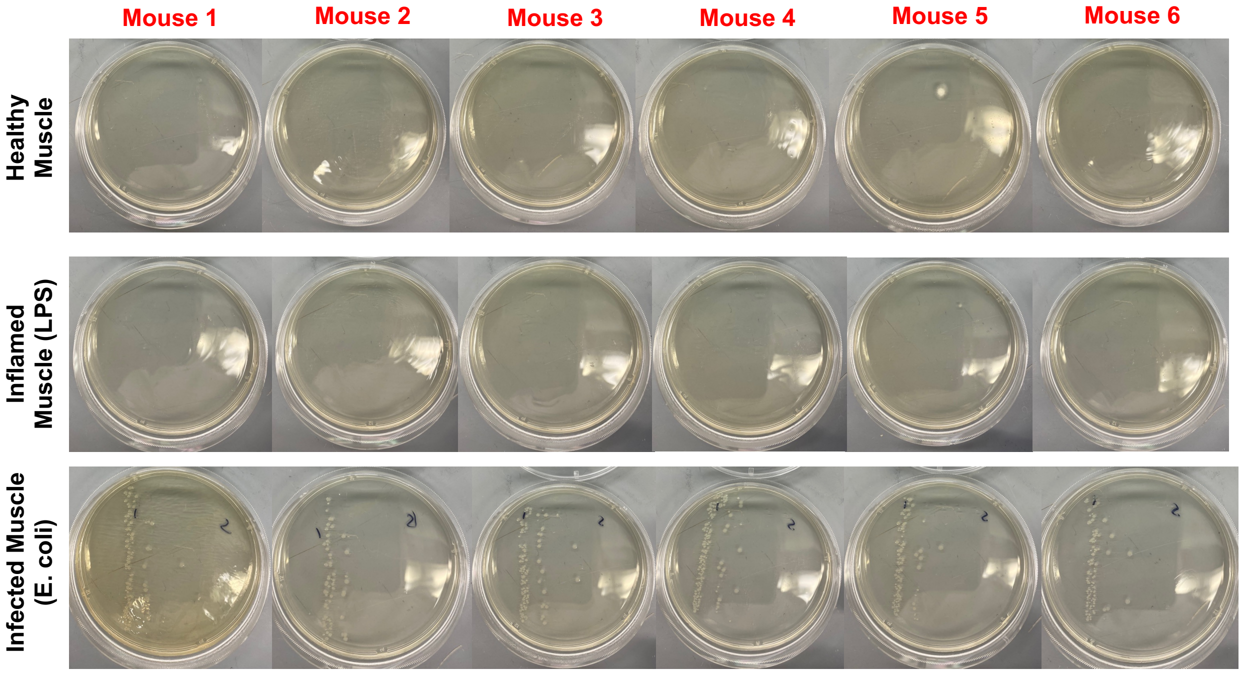

Figure S23. Growth of bacterial colonies on LB-agar plates inoculated with the homogenization products derived from *E. coli* K12 infected muscle tissue, inflamed muscle tissue (LPS induced), and healthy muscle harvested from mice in the cohort used for PET/CT imaging and *ex vivo* biodistribution studies with **[^{68}Ga]Ga^{III}-TREN-CAM**. Viable bacterial colonies are only observed in the plates inoculated with the *E. coli* K12 infected sample tissues.

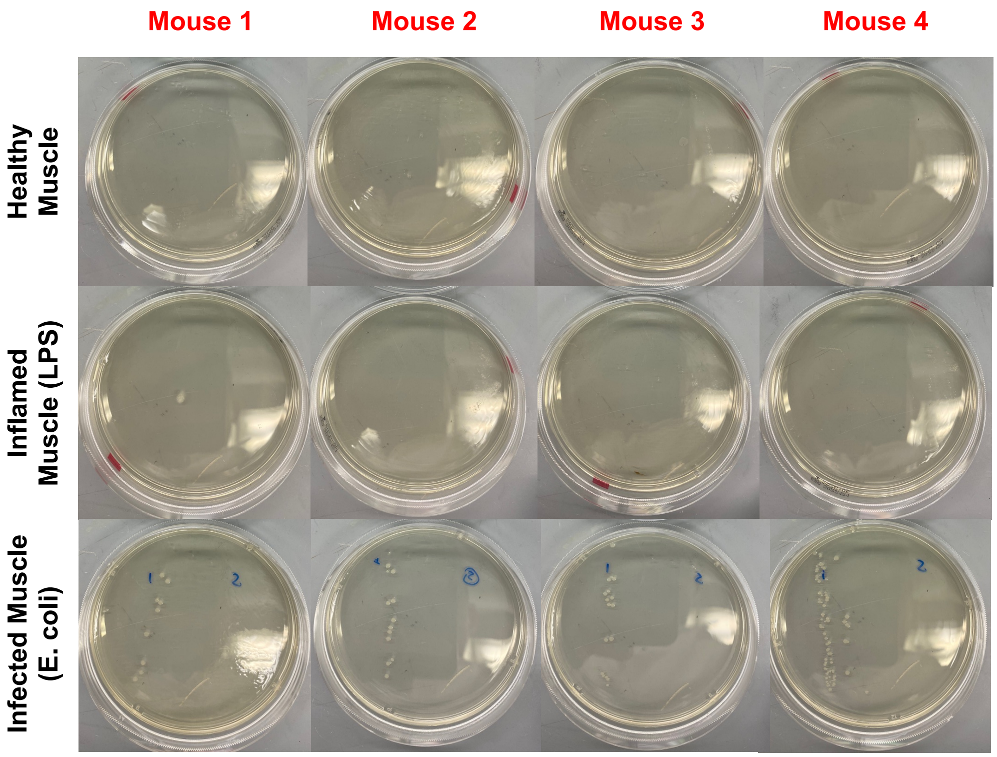

Figure S24. Growth of bacterial colonies on LB-agar plates inoculated with the homogenization products derived from *E. coli* K12 infected muscle tissue, inflamed muscle tissue (LPS induced), and healthy muscle harvested from mice in the cohort used for PET/CT imaging and *ex vivo* biodistribution studies with **[^{68}Ga]Ga^{III}-citrate**. Viable bacterial colonies are only observed in the plates inoculated with the *E. coli* K12 infected sample tissues

5.5 Assessment of Cefiderocol treatment in an *E. coli* K12 infected mice via PET-CT using [^{68}Ga]Ga^{III}-TREN-CAM as radiotracer

E. coli K12 bacterial inoculum was prepared as described in section 5.3.1.

Female balb/c mice were anesthetized with isoflurane and a 60 µL aliquot of bacterial inoculum (1.2 - 1.6 x 10^8 CFU/mL) was injected in the right triceps of the mouse (infected muscle), followed by administration of LPS (30 µL of LPS at 0.9 mg/mL) in the left triceps to provide a contralateral sterile inflammation control (inflamed muscle). Two cohorts were employed for the study; one cohort treated with the antibiotic cefiderocol (n = 6) and an untreated cohort (n = 6).

5.5.1 PET/CT imaging, Antibiotic Treatment, ROI analysis.

<u>*PET/CT Imaging Day 1 and cefiderocol treatment administration*</u>

Twenty-four hours after injection of the *E. coli* K-12 inoculum, 100 µL of a PBS formulation of [^{68}Ga]Ga^{III}-TREN-CAM (120–201 µCi) was administered via tail vein injection to four representative mice from each cohort. This selection was made because the PET/CT scanner was capable of imaging a maximum of four specimens simultaneously.

PET-CT scans were collected 1-hour post-injection of the radiotracer (25 hours post initial inoculation with *E. coli* K12). Scans were acquired in a Mediso nanoScan® PET/CT Scanner. Mice were initially

anesthetized with isoflurane gas scans at 2% isoflurane in oxygen and maintained during scans at 1.5%. PET-CT images were normalized to units of percentage of injected dose per cubic centimeter and presented as maximum intensity projected scan (MIPS) images.

Cefiderocol treatment or control (no treatment) doses were administered to all animals in their respective cohorts, including rodents not used for imaging, immediately after completion of PET/CT scans (26 h post-inoculation with *E. coli* K-12). Cefiderocol was administered subcutaneously (s.c) over the shoulders into the loose skin over the neck as a single dose of 40 mg/kg in 0.1 mL at 26 h post initial infection with *E. coli* K12. Treatment or control was administered while rodents were still under the effects of anesthesia post PET/CT imaging.

Antibiotic doses were prepared in 10% DMSO in water. Control (no treatment) doses correspond to 10% DMSO in water.

PET/CT Imaging Day 2

Forty-eight hours post injection of the *E. coli* K-12 inoculum, [^{68}Ga]Ga^{III}-TREN-CAM was administered to the same four mice imaged on Day 1 day to assess the progression of the bacterial infection in the untreated and cefiderocol-treated cohorts. PET-CT scans and images were acquired as previously described.

Two-dimensional (2D) images and maximum intensity projections scans (MIPs) were analyzed, and the region of interest (ROI) was manually segmented using Imalytics preclinical image analysis software. ROI analysis of the PET/CT images was used to estimate tracer uptake in major organs or tissues, and quantitative results are given as percentage of injected dose per cubic centimeter (%ID/cc).

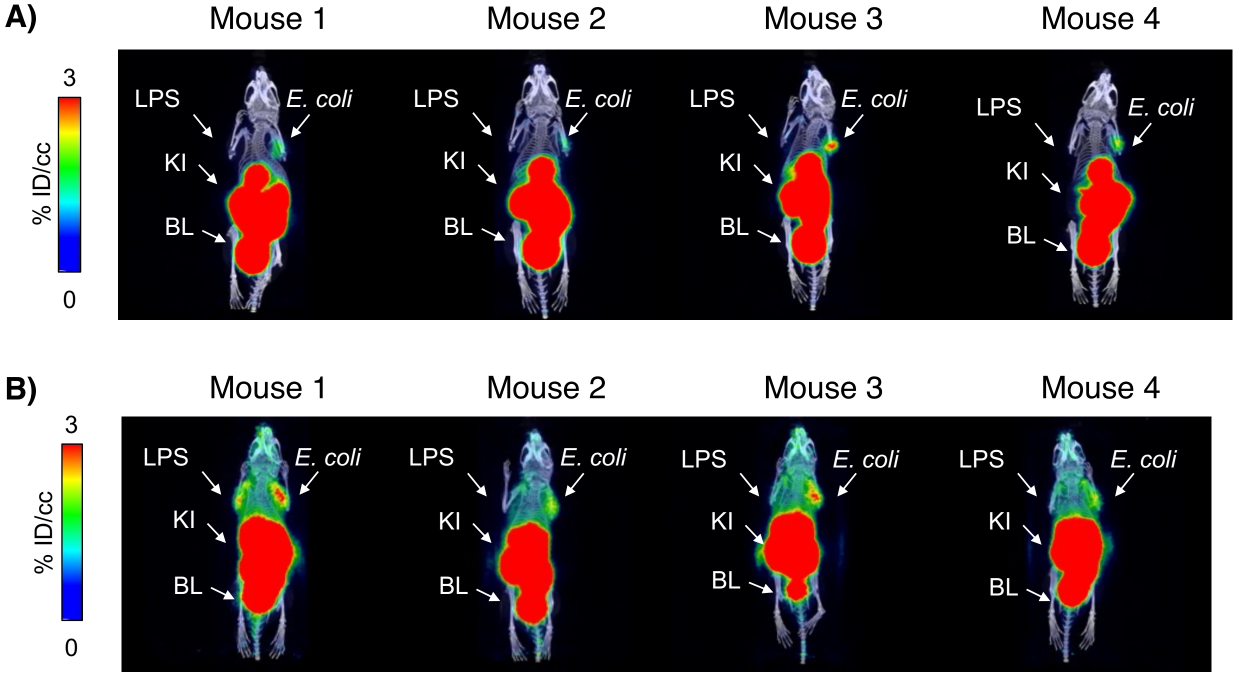

Figure S25. PET/CT images of [^{68}Ga]Ga^{III}-TREN-CAM in untreated BALB/c mice bearing an *E. coli* K12 infection. Images are normalized to units of %ID/cc and presented as maximum intensity projection scans (MIPS). Arrows point to organs of major interest: infected muscle (*E. coli*), inflamed muscle (LPS), Kidneys (Ki), and bladder (BL). The % ID/cc is the percentage of injected dose per cubic centimeter. A) Scans collected on Day 1, 1-hour postinjection of radiotracer (25 hours post inoculation with *E. coli* K12).

B) Scans collected on Day 2, 1-hour postinjection of radiotracer (49 hours post inoculation with *E. coli* K12).

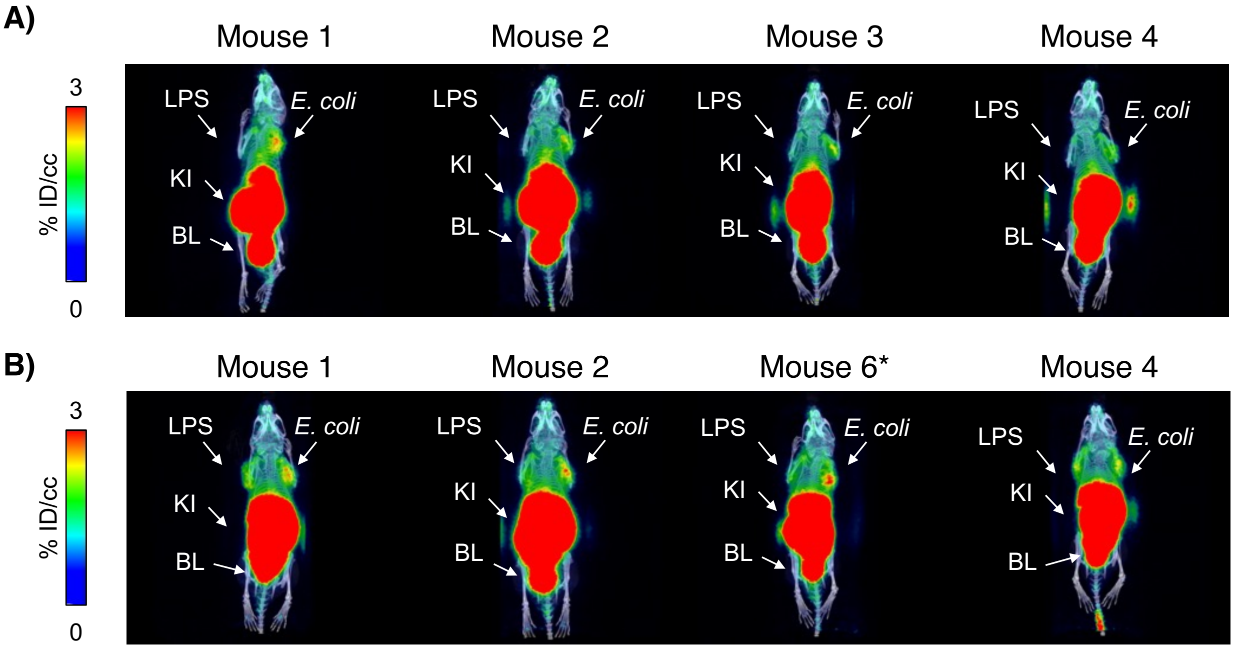

Figure S26. PET/CT images of [^{68}Ga]Ga^{III}-TREN-CAM in cefiderocol-treated BALB/c mice bearing an *E. coli* K12 infection. Images are normalized to units of %ID/cc and presented as maximum intensity projection scans (MIPS). Arrows point to organs of major interest: infected muscle (*E. coli*), inflamed muscle (LPS), Kidneys (Ki), and bladder (BL). The % ID/cc is the percentage of injected dose per cubic centimeter. A) Scans collected on Day 1, 1-hour postinjection of radiotracer (25 hours post inoculation with E. coli K12). B) Scans collected on Day 2, 1-hour postinjection of radiotracer (49 hours post inoculation with E. coli K12).

Table S8. Quantitative analysis of ROI segmentations for infected triceps muscle, inflamed triceps muscle, heart and kidneys for PET/CT scans acquired before antibiotic treatment initiation, corresponding to 25 hours post inoculation with *E. coli* K12. Results are given as percentage of injected dose per cubic centimeter (%ID/cc).

	**Cefiderocol Treated**				**Non-treated**			
Tissue	*Mouse 1*	*Mouse 2*	*Mouse 3*	*Mouse 4*	*Mouse 1*	*Mouse 2*	*Mouse 3*	*Mouse 4*
Infect. Musc	1.77	1.58	1.46	1.49	1.40	1.27	1.43	1.15
Infl. Musc	1.07	0.86	1.01	0.89	1.24	0.82	0.78	0.54
Healthy Musc	0.51	0.42	0.45	0.26	0.73	0.68	0.45	0.20
Heart	1.74	1.73	1.72	1.55	1.78	1.44	1.64	1.30
Kidney	75.01	73.55	76.63	67.26	63.93	58.53	70.93	59.11

Table S9. Quantitative analysis of ROI segmentations for infected triceps muscle, inflamed triceps muscle, heart and kidneys for PET/CT scans acquired post administration of antibiotic (treated cohort), corresponding to 49 hours post initial inoculation with *E. coli* K12. Results are given as percentage of injected dose per cubic centimeter (%ID/cc).

	**Cefiderocol Treated**				**Non-treated**			
Tissue	*Mouse 1*	*Mouse 2*	*Mouse 6*	*Mouse 4*	*Mouse 1*	*Mouse 2*	*Mouse 3*	*Mouse 4*
Infect. Musc	1.43	1.73	1.67	1.50	1.89	1.63	1.74	1.54
Infl. Musc	1.58	1.23	1.31	1.41	1.59	0.88	0.95	0.88
Healthy Musc	0.53	0.47	0.38	0.24	0.90	0.28	0.30	0.34
Heart	1.54	1.55	1.59	1.43	1.80	1.39	1.77	1.37
Kidney	37.68	39.82	32.74	32.21	26.73	21.02	40.75	28.07

5.5.2 Ex vivo biodistribution

Following PET-CT imaging on day 2, mice (n= 6 per cohort) were euthanized 2.0 hours post-injection of radiotracer (50 hours post initial inoculation with *E. coli* K12). Organs were harvested and biodistributions studies were conducted as previously described in section 5.3.3.

Table S10. Tabulated values for the biodistribution data of [^{68}Ga]Ga^{III}-TREN-CAM in *E. coli* K12 infected mice, with and without Cefiderocol treatment, at 2 hours post-injection of radiotracer (50 hours post infection. Values expressed as % ID/g. Error expressed as ± 1 SD for n = 6.

**Organs**	**[^{68}Ga]Ga^{III}-TREN-CAM Cefiderocol Treated Cohort**	**[^{68}Ga]Ga^{III}-TREN-CAM Non-treated Cohort**	**P value**
Blood	1.67 ± 0.56	1.43 ± 0.19	0.351194
Heart	0.68 ± 0.17	0.80 ± 0.08	0.161195
Lungs	1.59 ± 0.57	1.74 ± 0.21	0.564047
Liver	10.30 ± 4.86	13.55 ± 2.91	0.197668
Gallbladder	13.40 ± 6.26	23.27 ± 7.72	0.044092
Spleen	3.91 ± 1.65	5.65 ± 1.37	0.075715
Stomach	0.46 ± 0.20	0.97 ± 0.66	0.122711
Kidney	104.46 ± 15.45	98.07 ± 24.59	0.603788
Small Intestine	2.00 ± 1.35	3.55 ± 1.82	0.127077
Large Intestine	6.55 ± 3.70	6.23 ± 4.09	0.889480
Bone	1.02 ± 0.33	1.19 ± 0.22	0.326290
Infected Muscle	1.39 0.53	1.86 ± 0.25	0.088906
Inflamed Muscle	1.29 ± 0.59	1.04 ± 0.50	0.448045
Healthy Muscle	0.31 ± 0.09	0.37 ± 0.06	0.184329

5.5.3 Tissue collection and bacterial density assessment

Muscle tissues from the infected right triceps, inflamed left triceps, and healthy quadriceps were collected and treated as described in section 5.4.3.

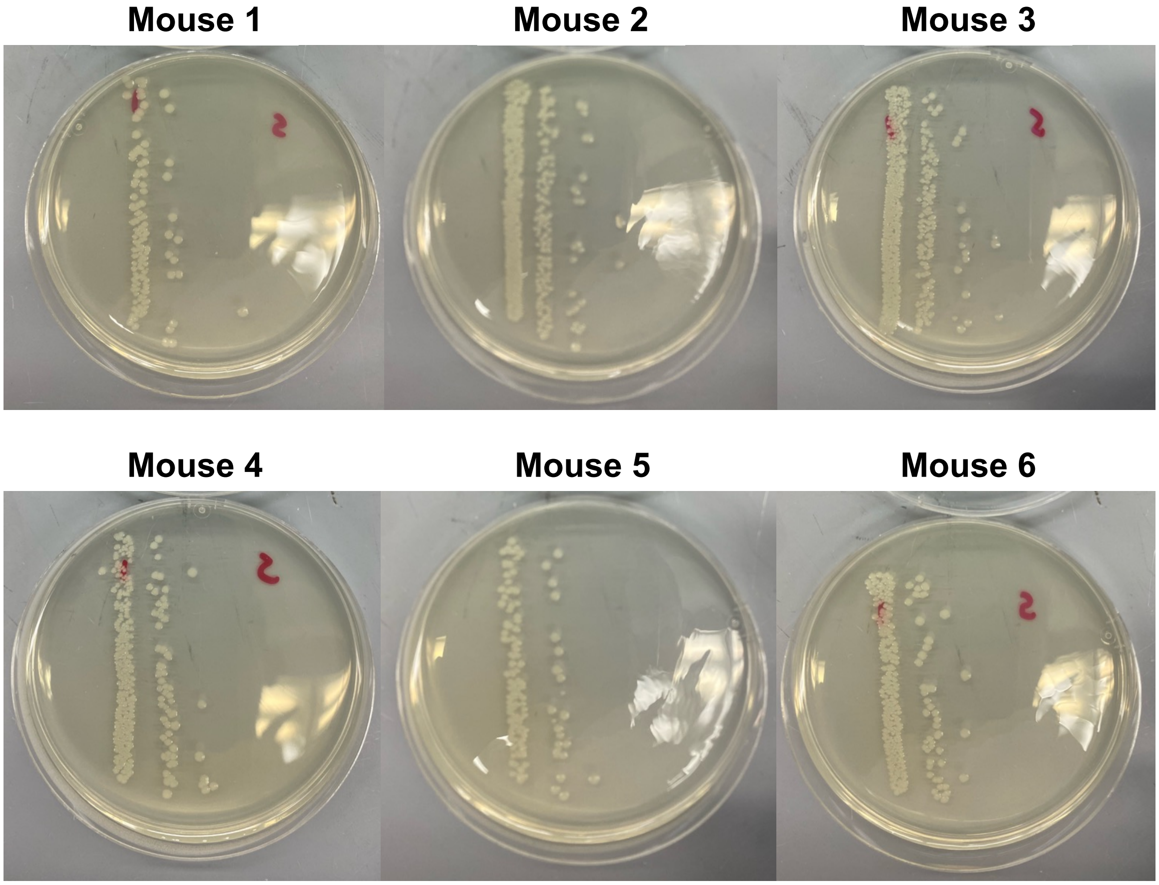

Figure S27. Growth of bacterial colonies on LB-agar plates inoculated with homogenization products derived from infected triceps muscle tissue from *E. coli* K12 infected mice treated with a single dose of cefiderocol (n=6). Each plate was inoculated with ten-fold serial dilutions of the original homogenate (5 dilutions per plate).

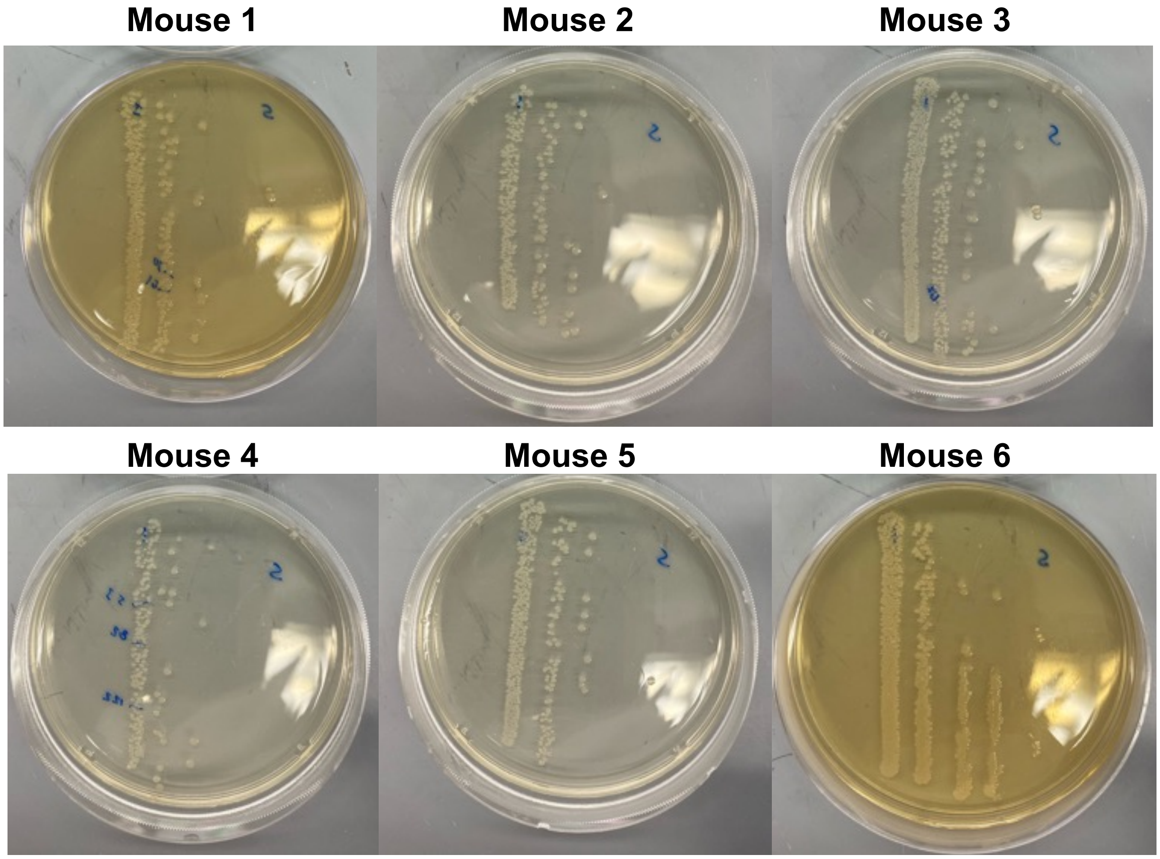

Figure S28. Growth of bacterial colonies on LB-agar plates inoculated with homogenization products derived from infected triceps muscle tissue from *E. coli* K12 infected mice (n=6) that did not receive cefiderocol treatment. Each plate was inoculated with ten-fold serial dilutions of the original homogenate (5 dilutions per plate).

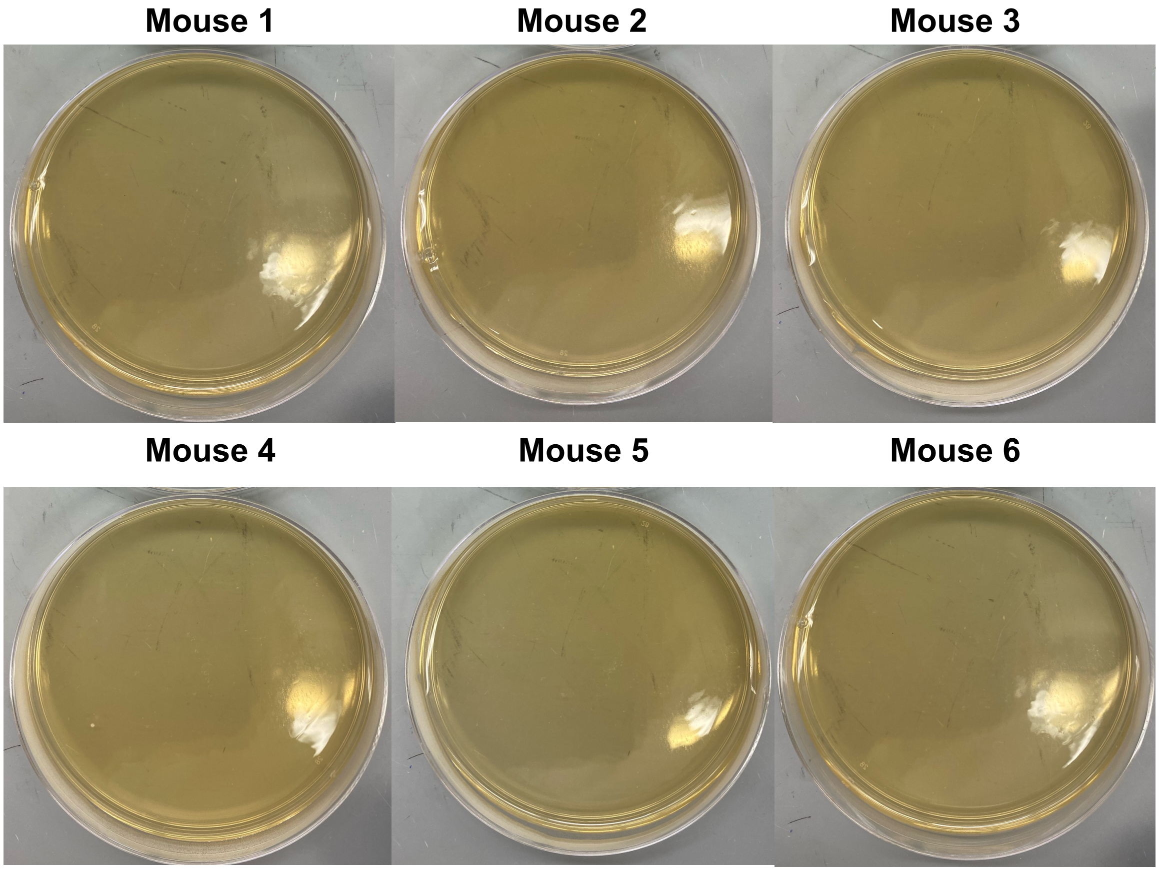

Figure S29. Absence of bacterial colonies on LB-agar plates inoculated with homogenization products derived from triceps muscle tissue inoculated with LPS. Muscle tissue recovered from *E. coli* K12 infected mice treated with a single dose of cefiderocol (n=6).

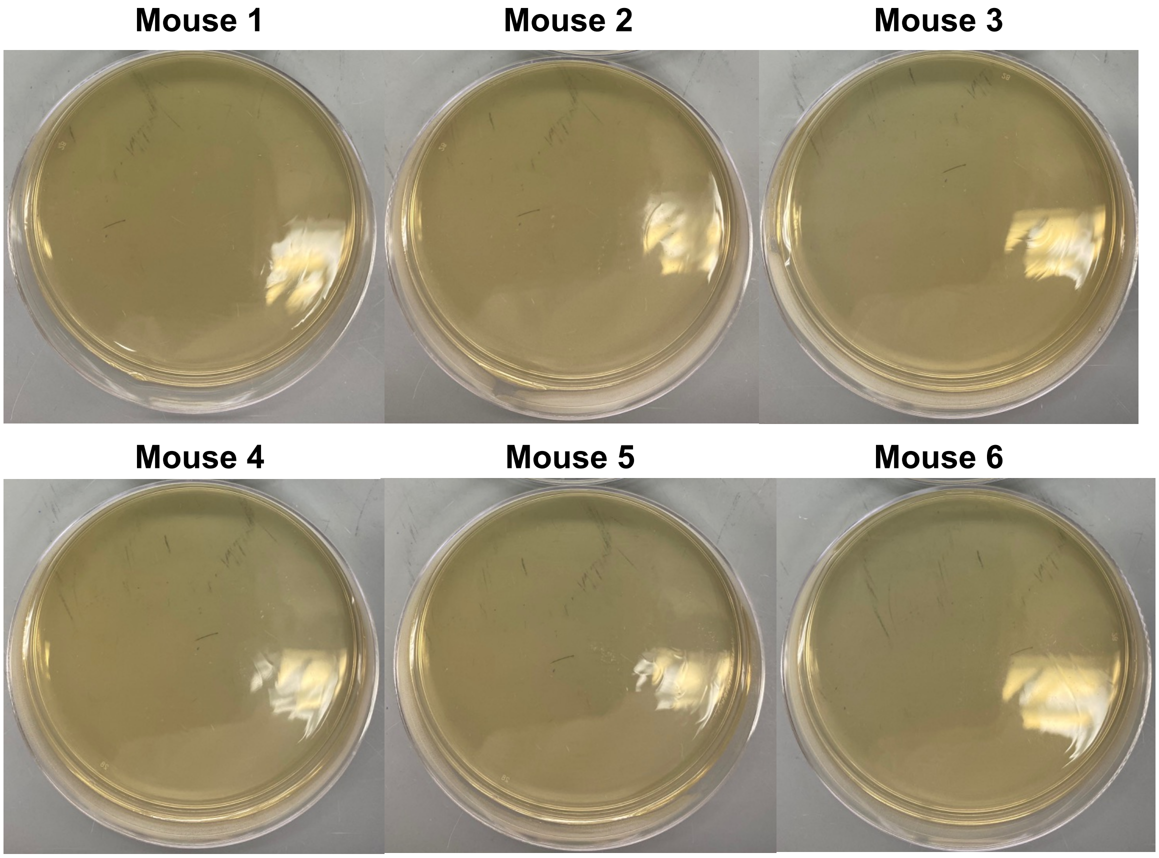

Figure S30. Absence of bacterial colonies on LB-agar plates inoculated with homogenization products derived from triceps muscle tissue inoculated with LPS. Muscle tissue recovered from *E. coli* infected mice (n=6) that did not receive cefiderocol treatment.

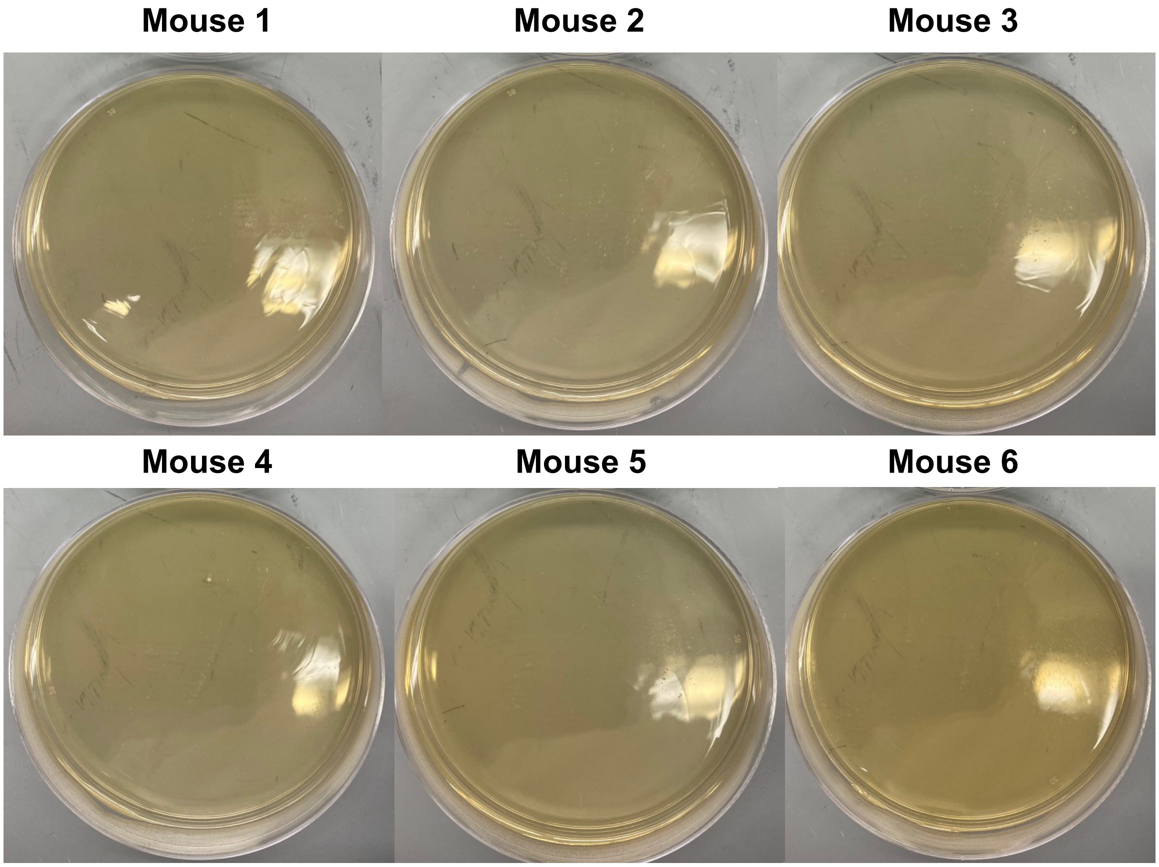

Figure S31. Absence of bacterial colonies on LB-agar plates inoculated with homogenization products derived from healthy muscle tissue recovered from *E. coli* K12 infected mice (n=6) that received a single dose of cefiderocol.

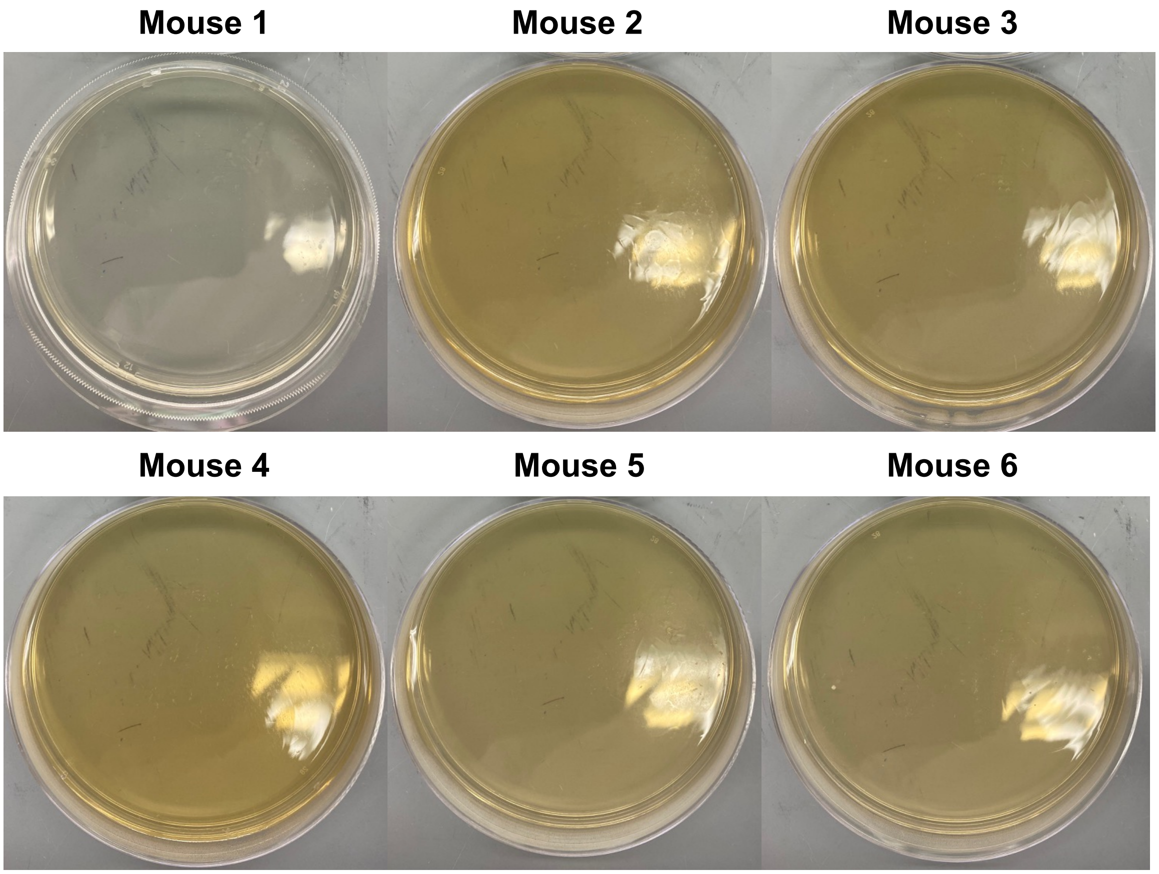

Figure S32. Absence of bacterial colonies on LB-agar plates inoculated with homogenization products derived from healthy muscle tissue recovered from *E. coli* K12 infected mice (n=6) that did not received cefiderocol treatment.

Table S11. Tabulated values for bacterial burden found for *E. coli* K12 infected muscle from untreated and cefiderocol mice cohorts .

Mouse	Cefiderocol Treated CFU/g Muscle	Non-treated Cohort CFU/g Muscle
1	1.63×10^7	7.67×10^7
2	1.08×10^8	2.96×10^7
3	1.05×10^8	1.09×10^8
4	3.88×10^7	1.95×10^7
5	2.36×10^7	7.21×10^7
6	3.51×10^7	3.13×10^8
Average	$(5.44 \pm 3.75) \times 10^7$	$(1.03 \pm 0.98) \times 10^8$

References

[1] M. A. Joaqui-Joaqui, M. K. Pandey, A. Bansal, M. V. R. Raju, F. Armstrong-Pavlik, A. Dundar, H. L. Wong, T. R. DeGrado, V. C. Pierre, *Inorg. Chem.* **2020**, *59*, 12025-12038.

[2] A. Pandey, C. Savino, S. H. Ahn, Z. Yang, S. G. Van Lanen, E. Boros, *J. Med. Chem.* **2019**, *62*, 9947-9960.

[3] E. A. Dertz, J. Xu, K. N. Raymond, *Inorg. Chem.* **2006**, *45*, 5465-5478.

[4] R. Ben Azzouna, A. Guez, K. Benali, F. Al-Shoukr, W. Gonzalez, P. Karoyan, F. Rouzet, D. Le Guludec, *EJNMMI Radiopharm. Chem.* **2017**, *2*, 3.

[5] V. Ščasnár, J. E. van Lier, *Eur. J. Nucl. Med.* **1993**, *20*, 273-273.

[6] M. Petrik, A. Vlckova, Z. Novy, L. Urbanek, H. Haas, C. Decristoforo, *Biomedical papers* **2015**, *159*, 060-066.

[7] T. Ito-Horiyama, Y. Ishii, A. Ito, T. Sato, R. Nakamura, N. Fukuhara, M. Tsuji, Y. Yamano, K. Yamaguchi, K. Tateda, *Antimicrob. Agents Chemother.* **2016**, *60*, 4384-4386.
